# Altered expression of the L-arginine/nitric oxide pathway in ovarian cancer: metabolic biomarkers and biological implications

**DOI:** 10.1186/s12885-023-11192-8

**Published:** 2023-09-08

**Authors:** Linfeng Chen, Qiming Tang, Keying Zhang, Qianyang Huang, Yun Ding, Bo Jin, Szumam Liu, KuoYuan Hwa, C. James Chou, Yani Zhang, Sheeno Thyparambil, Weili Liao, Zhi Han, Richard Mortensen, James Schilling, Zhen Li, Robert Heaton, Lu Tian, Harvey J. Cohen, Karl G. Sylvester, Rebecca C. Arent, Xinyang Zhao, Doff B. McElhinney, Yumei Wu, Wenpei Bai, Xuefeng B. Ling

**Affiliations:** 1 Beijing Shijitan Hospital, Capital Medical University, Beijing, China; 2 Shanghai Yunxiang Medical Technology Co., Ltd., Shanghai, China; 3 Binhai Industrial Technology Research Institute, Zhejiang University, Tianjin, China; 4 Beijing Obstetrics and Gynecology Hospital, Capital Medical University, Beijing Maternal and Child Health Care Hospital, Beijing, China; 5 mProbe Inc, Palo Alto, CA USA; 6 Tianjin Yunjian Medical Laboratory Institute Co., Ltd, Tianjin, China; 7 School of Medicine, University of Alabama at Birmingham, Birmingham, AL USA; 8 School of Medicine, Stanford University, Stanford, CA USA

**Keywords:** Arginase (ARG), Nitric oxide synthase (NOS), Dimethylarginine dimethylaminohydrolase (DDAH), Protein arginine methyltransferases (PRMT), Asymmetric dimethylarginine (ADMA), Symmetric dimethylarginine (SDMA), Dimethylamine (DMA), Metabolic reprogramming, Ovarian cancer (OC), L-arginine/nictric oxide (L-ARG/NO)

## Abstract

**Motivation:**

Ovarian cancer (OC) is a highly lethal gynecological malignancy. Extensive research has shown that OC cells undergo significant metabolic alterations during tumorigenesis. In this study, we aim to leverage these metabolic changes as potential biomarkers for assessing ovarian cancer.

**Methods:**

A functional module-based approach was utilized to identify key gene expression pathways that distinguish different stages of ovarian cancer (OC) within a tissue biopsy cohort. This cohort consisted of control samples (*n* = 79), stage I/II samples (*n* = 280), and stage III/IV samples (*n* = 1016). To further explore these altered molecular pathways, minimal spanning tree (MST) analysis was applied, leading to the formulation of metabolic biomarker hypotheses for OC liquid biopsy. To validate, a multiple reaction monitoring (MRM) based quantitative LCMS/MS method was developed. This method allowed for the precise quantification of targeted metabolite biomarkers using an OC blood cohort comprising control samples (*n* = 464), benign samples (*n* = 3), and OC samples (*n* = 13).

**Results:**

Eleven functional modules were identified as significant differentiators (false discovery rate, FDR < 0.05) between normal and early-stage, or early-stage and late-stage ovarian cancer (OC) tumor tissues. MST analysis revealed that the metabolic L-arginine/nitric oxide (L-ARG/NO) pathway was reprogrammed, and the modules related to "DNA replication" and "DNA repair and recombination" served as anchor modules connecting the other nine modules. Based on this analysis, symmetric dimethylarginine (SDMA) and arginine were proposed as potential liquid biopsy biomarkers for OC assessment. Our quantitative LCMS/MS analysis on our OC blood cohort provided direct evidence supporting the use of the SDMA-to-arginine ratio as a liquid biopsy panel to distinguish between normal and OC samples, with an area under the ROC curve (AUC) of 98.3%.

**Conclusion:**

Our comprehensive analysis of tissue genomics and blood quantitative LC/MSMS metabolic data shed light on the metabolic reprogramming underlying OC pathophysiology. These findings offer new insights into the potential diagnostic utility of the SDMA-to-arginine ratio for OC assessment. Further validation studies using adequately powered OC cohorts are warranted to fully establish the clinical effectiveness of this diagnostic test.

**Supplementary Information:**

The online version contains supplementary material available at 10.1186/s12885-023-11192-8.

## Introduction

Ovarian cancer (OC) accounting for over 21,410 new cases and around 13,770 deaths in the US in 2021 [1] is the leading cause of deaths among woman cancers and has the poorest prognosis among gynecological malignancies. The high OC mortality rate is related to the fact that approximately three fourths of women have stage III or IV disease at diagnosis. Because of the absence of distinct early symptoms, it is difficult to diagnose asymptomatic localized-stage early cancer when the tumor is still confined to the ovary and potentially curable by debulking surgery and chemotherapy [2].

The current most widely used screening tool is the combination of the cancer antigen 125 (CA125) blood test and transvaginal ultrasonography (TVUS). However, neither multimodal screening (MMS) nor transvaginal ultrasound screening approaches have significantly reduced deaths from ovarian cancer [3]. HE4 was by the U.S. Food and Drug Administration (FDA) as a monitoring method for ovarian cancer patient management [4]. The five-year ovarian cancer survival rate has not been improved over the past three decades [5]. Clearly, from the molecular diagnostics perspective, identification of novel biomarkers to allow early detection of new onset disease, and routine surveillance of minimal residual disease (MRD) from which some patients experience recurrent disease after a curative-intent treatment, should be essential to achieve better outcomes in OC patients.

The last decade has witnessed increasingly rapid advances in multiple omics technologies [6], including genomics, transcriptomics, proteomics, and metabolomics. Multi-omics based deep molecular profiling has been widely applied to analyze tissue- and liquid-derived samples from OC patients. The integration of high dimensional datasets has increased our knowledge of the disease and improved our understanding of the molecular landscape of OC. Current work in elucidating relationships between different stages or subtypes of cancer has largely been based on pre-existing knowledge of cancer associated genes. Quantitative network-based framework has been previously established to compare diseases by an integrated analysis of disease-related mRNA express data and molecular pathological modules [7]. Common or discriminant functional modules and processes can be identified to discover the disease-disease similarities or differences. Novel hypotheses for disease pathophysiology can thus be proposed to understand the underlying biology of the observed disease correlations.

Although tumor tissue biopsy profiling has been the standard for evaluating molecular cancer features, it is invasive and difficult to obtain serially. Therefore, liquid biopsy has emerged as a promising approach with several unique advantages for cancer diagnosis. First, it is minimally invasive and safe, avoiding the potential complications caused by tissue biopsy. Second, it provides an opportunity to identify heterogeneous tumor-specific alterations that may be missed by tissue biopsy [8]. More importantly, liquid biopsy enables serial sampling over time, which provides important information for guiding clinical decisions. Still, the cancer field is short of sensitive and specific blood biomarkers to aid diagnosis and clinical decision making or to serve as molecular targets for chemoprevention and treatment.

In this study, we set to use a tissue biopsy-based module analysis approach to identify aberrant cancer metabolic pathways and develop metabolite biomarker hypotheses for OC assessment. To translate cancer metabolic abnormalities into clinical diagnostics, a liquid biopsy based quantitative mass spectrometry method was developed to validate these metabolite biomarkers’ potential clinical utilities in OC care.

## Materials and methods

### Blood collection and ethical considerations: ensuring responsible practices in human research

The study of blood samples from ovarian subjects was conducted in accordance with ethical guidelines and approved by the Institutional Review Board of Beijing Shijitan Hospital, Capital Medical University. The study adhered to the principles outlined in the Declaration of Helsinki. All experimental procedures were carried out in compliance with the requirements of the Human Ethics Procedures and Guidelines set by the local government. Prior to participating in the study, written informed consent was obtained from all participants. The plasma samples were collected at the time of diagnosis, prior to any treatment, and comprised 464 samples from individuals with normal conditions, 3 samples from patients with benign tumors, and 13 samples from OC patients (Table 1). To obtain serum samples, collected blood was allowed to clot at room temperature for 30 min. Subsequently, the samples underwent centrifugation at 3000 r/min for 5 min. After centrifugation, all serum samples were divided into smaller aliquots and stored at a temperature of -80ºC until further use.

**Table 1:**
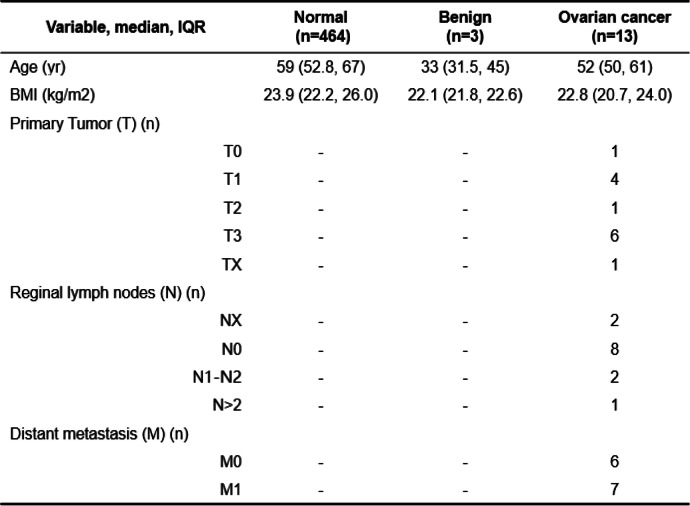
Demographics of the pilot validation cohort

### Research strategy and workflow: a comprehensive approach for investigating tissue and liquid biopsy biomarkers assessing ovarian cancer

**Fig. 1:**
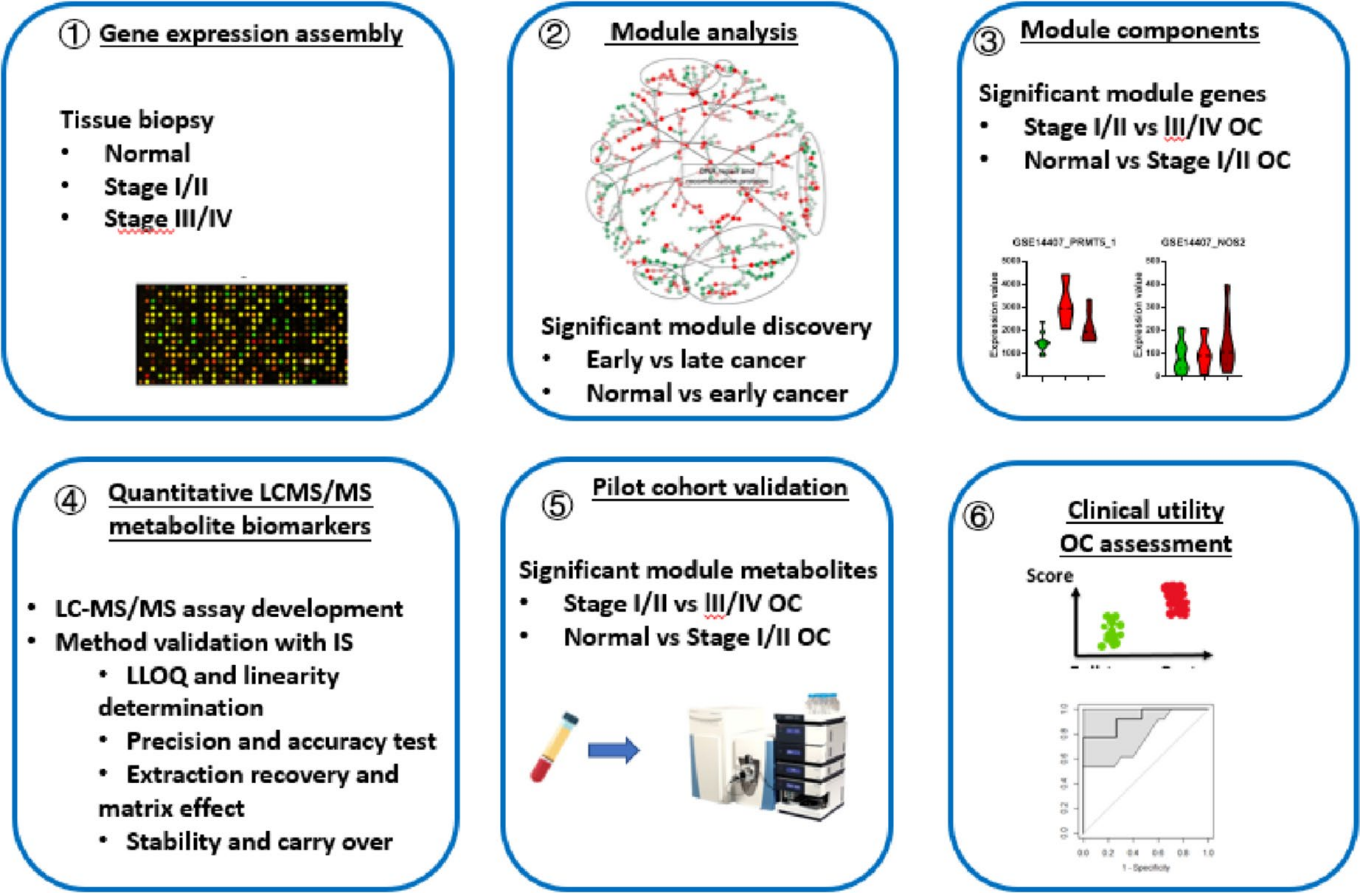
Study workflow diagram. OC: ovarian cancer. Overall, our study employed a comprehensive approach involving gene expression profiling, functional module analysis, targeted gene characterization, metabolite biomarker validation, and the development of a metabolite panel. This methodology aimed to identify and validate potential biomarkers for different stages of ovarian cancer

Our study followed a systematic approach consisting of six steps, as depicted in Fig. 1. Firstly, we collected gene expression profiling datasets comprising both normal ovarian tissues and tissues from different stages of ovarian cancer (Supplementary data 2 Table 1). Secondly, we conducted functional module analysis to identify modules that exhibited significant differences between normal ovarian tissue and early-stage ovarian cancer, as well as between early-stage and late-stage ovarian cancer tissues. Thirdly, we characterized the gene expression patterns of the targeted module component genes, specifically focusing on their differentiation between normal ovarian tissue and early-stage ovarian cancer, as well as between early-stage and late-stage ovarian cancer tissues. These two steps aided in the formulation of hypotheses regarding ovarian cancer biomarkers, which could be based on either the functional modules or the targeted module component genes.

Additionally, we explored the targeted functional modules, module component genes, and the associated substrate/product metabolites to propose the possibility of utilizing these metabolites as mechanism of action (MOA)-derived ovarian cancer biomarkers. This analysis further expanded our understanding of the potential underlying mechanisms of ovarian cancer.

In the fourth step, we developed quantitative LC–MS/MS biomarker assays to validate the metabolite biomarker hypotheses in blood samples. Subsequently, in the fifth step, we validated the levels of these metabolite biomarkers in a pilot cohort of blood samples. Finally, in the sixth step, we developed a metabolite panel that allowed for the detection and differentiation of various stages of ovarian cancer.

### Identification and analysis of gene expression functional modules

Functional module analysis [7] as conducted to compare gene expression patterns between (1) normal ovarian tissue and early-stage ovarian cancer, and (2) early-stage ovarian cancer and late-stage ovarian cancer. All genes were annotated to the KEGG metabolic pathways and our curated biological modules. Gene expression datasets were quantile normalized to ensure comparability. To assess the statistical significance of differential gene expression, Mann–Whitney U test statistics were computed for each gene, comparing (A) normal ovarian tissue versus stage I/II cancer, and (B) stage I/II cancer versus stage III/IV cancer. When comparing U test analysis results from different datasets, it was necessary to adjust the U test statistic based on the sample sizes of the compared groups. Specifically, for gene $i$ in case group $(with\ a\ group\ size\ of\ n1)$ and control group $(with\ a\ group\ size\ of\ n2)$, the statistic [9] corresponding with the control group was computed as $U{2}_{i}=\mathrm{n}1\cdot \mathrm{n}2-{U1}_{i}$, where ${U1}_{i}$ represents the statistic corresponding to the case group. To perform significance analysis for both case and control groups, a derived gene score was computed as follows:

$${\mathrm{Gene\ Score }}_{\mathrm{i}}=\frac{{\mathrm{U}1}_{\mathrm{i}}-{\mathrm{U}2}_{\mathrm{i}}}{\mathrm{n}1+\mathrm{n}2}=\frac{2\cdot {\mathrm{U}1}_{\mathrm{i}}-\mathrm{n}1\cdot \mathrm{n}2}{\mathrm{n}1+\mathrm{n}2}$$

This gene score represented the significance of gene i in distinguishing between the two compared groups. Furthermore, a functional module score [7] was calculated as the median of the "Gene Scores" of the component genes within each functional module. To evaluate the significance of the functional module score, false discovery analysis [10, 11] was conducted. This involved permutating samples within the gene expression matrices 100 times to estimate the false discovery rate.

### Gene expression correlation network analysis

To visually represent the significant functional module component genes, a graph structure was employed. Each gene was depicted as a node within the graph. The correlation structure among these genes was extracted using a Minimum Spanning Tree (MST) approach. In the MST, the widths of the edges connecting the nodes were determined based on the Spearman *p* value, representing the correlation strength between the respective gene pairs. These edge widths were represented on a logarithmic scale, emphasizing the significance of the correlations.

By utilizing this graph-based representation, the interconnections and relationships between the significant functional module component genes could be visually interpreted. The width of the edges provided an indication of the strength of the correlations, allowing for a comprehensive understanding of the gene–gene interactions within the module.

### Optimized sample preparation for accurate metabolite quantification

For the extraction process, 10 μL of the calibrator, quality control (QC), or unknown sample was mixed with 100 μL of 10% trichloroacetic acid (TCA) and 100 μL of the internal standard (IS) working solution containing 2.5 μM of ^13^C_6_-arginine and D^7^-ADMA. To ensure thorough mixing, the extracts were vortexed for 30 s. Subsequently, the samples were centrifuged at 12,000Xg under 4 °C for 3 min. After centrifugation, 180 μL of the supernatant was carefully collected from each sample and transferred into an auto-sampler vial equipped with a micro-insert. The purpose of transferring the supernatant into the auto-sampler vial was to prepare the samples for subsequent analysis using liquid chromatography/mass spectrometry (LC/MS). This step allowed for the proper handling and introduction of the samples into the LC/MS system.

### Quantitative LC/MS/MS analysis of targeted metabolites

The Dionex Ultimate 3000 UHPLC system utilized in the analysis comprised various components from Thermo Fisher, including a degasser, an RS binary pump, an RS auto-sampler, and an RS column compartment located in San Jose, CA. To interface with the UHPLC system, a TSQ Quantiva mass spectrometer equipped with an electrospray ionization source and a built-in Rheodyne switch valve from Thermo Fisher was employed. Data acquisition and chromatographic peak integration were carried out using the XCalibur 4.0 software package, also from Thermo Fisher.

After the sample preparation process, 10 μL of the prepared sample was injected onto an ACE Excel SuperC18 column (1.7 mm, 50 mm × 2.1 mm; MAC-MOD Analytical, Chadds, PA). The mobile phases consisted of water with 0.1% formic acid (A) and methanol with 0.1% formic acid (B). Chromatographic separation was achieved using a 2-min isocratic elution at a flow rate of 0.3 mL/min with 5% B. Initially, the LC eluent was directed to waste for the first minute, and subsequently switched back to the electrospray interface from 1.1 to 2.0 min, enabling the elution, ionization, and detection of the targeted analytes by the system. Throughout the analysis, the auto-sampler and column oven temperatures were maintained at 4 °C and 30 °C, respectively.

Operating in a multiple reaction monitoring (MRM) mode, the mass spectrometer acquired data from the LC eluent. The MRM transitions for the targeted analytes were individually optimized by infusing 2.5 mM of commercial standards at a rate of 10 mL/min into the mass spectrometer in the presence of 0.5% formic acid. The optimized MRM transitions can be found in Table 2A, provided for both the individual analytes and internal standards. The Q1 and Q3 resolutions were set at 0.7 Da, and the cycle time was set at 0.5 s. Furthermore, the source parameters were optimized by mixing 5% B LC flow at 0.3 mL/min with a standard cocktail of 2.5 mM via a syringe pump infusion and a tee connector. The spray voltage was optimized at 3500 V, while the optimal gas flows for Sheath Gas, Aux Gas, and Sweep Gas were determined to be 20, 5, and 0 Arb, respectively. The ion transfer tube and vaporizer temperatures were also optimized at 300 °C and 175 °C, respectively. The quantitation of the targeted analytes was carried out using a systematic approach. Initially, the chromatographic peak areas corresponding to the quantifier ions of the targeted analytes were integrated for all samples in the extracted ion chromatograms (EICs). Subsequently, the integrated peak areas were subjected to manual inspection. The area under the curve (AUC) for each analyte was then normalized by dividing it with the AUC of the corresponding internal standard (IS). Following the normalization step, the IS-normalized peak area ratios obtained from the calibrator samples were plotted against their respective concentrations. This process allowed the establishment of calibration curves based on six concentration levels. These calibration curves served as a reference to determine the concentrations of the targeted analytes in subsequent samples.

**Table 2:**
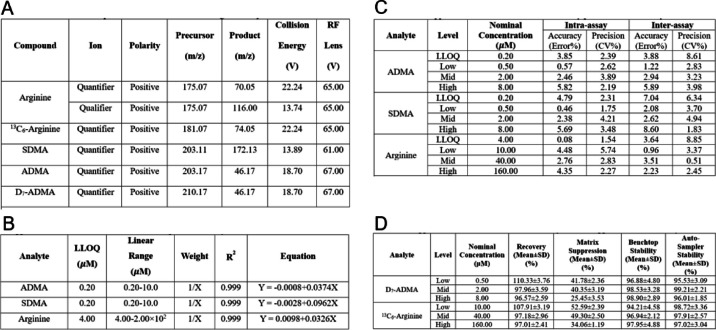
Presents the optimized quantitative mass spectrometry assays for the targeted analytes involved in the L-ARG/NO pathway, specifically arginine, SDMA, and ADMA. The table encompasses several key aspects of the assays as follows: (A) SRM Transitions: This section outlines the selected reaction monitoring (SRM) transitions for each analyte, providing the specific mass-to-charge ratio (m/z) values used for quantification. (B) LLOQ, Linear Range, and Linearity: Here, the table presents the lower limit of quantification (LLOQ), the linear range of concentrations, and the linearity of the calibration curves for the respective analytes. This information helps determine the sensitivity and dynamic range of the assays. (C) Intra- and Inter-Assay Precision and Accuracy: This section provides data on the precision and accuracy of the assays. It includes both intra-assay (within-run) precision and accuracy, as well as inter-assay (between-run) precision and accuracy. These values indicate the reliability and reproducibility of the measurements. (D) Extraction Recovery, Matrix Suppression, and Stability: The table also includes information on the extraction recovery, matrix suppression, and stability of the analytes. These parameters assess the efficiency of the sample extraction process, the impact of sample matrix on analyte detection, and the stability of the analytes during storage and analysis. By organizing these essential assay parameters here, it facilitates a comprehensive understanding of the optimized mass spectrometry assays for the targeted L-ARG/NO pathway analytes, aiding researchers in their analysis and interpretation of the data

Quantification of the targeted analytes involved employing linear regression fitting with a weighting factor of 1/x to construct the calibration curves. Subsequently, an acceptance cutoff was implemented to ensure the calibration quality, requiring the square of the correlation coefficient (r2) to be greater than 0.99. This stringent criterion ensured the reliability and accuracy of the calibration curves.

Following the establishment of the calibration curves, the IS-normalized peak area ratios were applied to these curves. By utilizing the calibration curves, absolute concentrations of the targeted analytes in both pooled and unknown serum samples were determined. This step allowed for the accurate quantification of the analytes based on their respective peak area ratios and the calibration curves generated. Overall, this approach provided a robust and validated method for obtaining precise and reliable absolute concentrations of the targeted analytes in the serum samples, facilitating accurate data analysis and interpretation in the study.

A pooled serum sample obtained from healthy volunteer subjects was utilized to determine the mean and standard deviations of endogenous concentrations for the targeted analytes through both intra- and inter-assay measurements. These measurements were crucial in establishing quality assurance criteria based on statistical principles.

To ensure batch acceptance, a specific procedure was followed. Pooled serum samples, comprising 10% of the unknown samples, were prepared and evenly distributed throughout the batch for injection. For batch acceptance, it was required that the imprecision and inaccuracy of the measurements obtained from the pooled serum samples be less than 15%. Additionally, mean response ratios of quantifier to qualifier ions were determined using commercial standards for each analyte. A criterion was set that individual serum samples within the batch should not deviate more than 15% from the mean response ratios in order to qualify. This approach, employing statistical criteria and quality assurance measures, allowed for the accurate characterization and qualification of individual serum samples within the batch, thereby ensuring the reliability and validity of the results.

### Development and validation of quantitative LC–MS/MS assays for L-arginine SDMA and ADMA

Aberrant metabolism is a prominent characteristic of cancer, displaying remarkable flexibility that is specific to cancer type and context. Consequently, our research endeavors to utilize quantitative metabolic profiling to validate biomarker hypotheses and effectively translate metabolic abnormalities into clinical practice.

The calibration standards for creatinine and arginine were procured from Sigma Aldrich, located in St. Louis, MO. As for the calibration standards for asymmetric dimethyl arginine (ADMA) and symmetric dimethyl arginine (SDMA), they were obtained from EMD Millipore, based in Burlington, MA. To ensure accurate measurements, stable isotope-labeled internal standards, specifically ^13^C_6_-L-arginine, and D_7_-ADMA, were purchased from Cambridge Isotope Laboratory in Tewksbury, MA. HPLC grade water, methanol, and formic acid, which are essential for the analytical process, were obtained from Fisher Scientific situated in Pittsburgh, PA. ACS grade trichloroacetic acid (TCA), an important reagent, was sourced from Sigma Aldrich. It is worth mentioning that all the materials were used directly without the need for additional purification.

To prepare the necessary solutions for the analysis, the following steps were taken. First, stock solutions of arginine and SDMA were prepared by dissolving the lyophilized powders in ddH_2_O to achieve a concentration of 10.0 mM. Next, a set of six-level calibrator working solutions was created by serially diluting the stock solutions with ddH_2_O. This resulted in concentrations of 0.20, 0.40, 1.00, 2.00, 5.00, and 10.0 µM for both ADMA and SDMA, and concentrations of 4.00, 8.00, 20.0, 40.0, 1.00 × 102, and 2.00 × 10^2^ µM for arginine. Similarly, a set of four-level quality control (QC) working solutions was prepared by serial dilutions of the stock solutions with ddH_2_O. This yielded concentrations of 0.20, 0.50, 2.00, and 8.00 µM for SDMA, and concentrations of 4.00, 10.00, 40.0, and 1.60 × 10^2^ µM for arginine. Additionally, a system suitability working solution was prepared by serial dilutions of the stock solutions with ddH_2_O. This resulted in a concentration of 0.33 µM for SDMA and 6.68 µM for arginine. Moving on to the internal standards, the stock solutions of ^13^C_6_-arginine, and D_7_-ADMA were prepared by dissolving the lyophilized powders in ddH_2_O to achieve a concentration of 10.0 mM. The internal standard (IS) working solution was then prepared by serial dilutions of the stock solutions with methanol, resulting in a concentration of 2.50 µM for each analyte. For the TCA solution, 50.0 g of TCA powder was weighed and dissolved in 22.7 mL of ddH2O to obtain a concentration of 100% (w/v) in the stock solution. The working solution of TCA was prepared by diluting the stock solution with ddH2O to achieve a concentration of 10% (w/v). All the prepared solutions were stored at 4 °C prior to their intended use in the analysis.

Table 2A presents the optimized multiple reaction monitoring (MRM) transitions for individual analytes and internal standards (ISs), encompassing both quantifier and qualifier transitions. To achieve optimal performance, Q1 and Q3 resolutions were set at 0.7 Da, while the cycle time was established at 0.5 s. Additionally, collision energy and RF lens settings for each analyte and ISs are provided for reference.

In Table 2B, the lower limit of quantitation (LLOQ) and linear range for each analyte are outlined. The finalized LLOQs were determined to be 4.00 µM for arginine and 0.20 µM for ADMA and SDMA. Importantly, all analytes exhibited excellent linearity, as evidenced by *R*^*2*^ values surpassing 0.99.

To evaluate precision and accuracy, both intra- and inter-assay measurements were performed. This involved analyzing quality control (QC) samples, prepared in a surrogate matrix, at four distinct concentrations: LLOQ, Low, Medium, and High. Each sample underwent analysis in six replicates across four independent runs. The results, presented in Table 2C, indicate that the coefficient of variation (CV%) for intra-assay measurements remained below 5.74%, while the percent error (PE%) was below 5.82%. Similarly, for inter-assay measurements, the CV% and PE% values were below 8.85% and 8.60%, respectively.

These meticulous assessments of precision and accuracy ensure the reliability and reproducibility of our analytical methodology when quantifying the targeted analytes. By adhering to stringent quality control measures, we establish the robustness of our results, further bolstering their potential applicability in clinical settings.

The evaluation of extraction recovery, which quantifies the percentage of the known analyte amount retained throughout the sample extraction and processing steps of the method, was conducted. Table 2D presents the results, revealing that the percent recoveries for all analytes at various concentrations exceeded 89.4%. This indicates minimal loss of analytes during the sample extraction and processing steps, ensuring the integrity of the measured concentrations.

Matrix effect and stability assessments were also performed. Table 2D demonstrates the percent signal suppressions, which ranged from 25 to 52% among different analytes. However, it is important to note that these suppressions remained highly consistent across replicates at different concentrations. This consistency strengthens the reliability and reproducibility of our analytical approach.

Regarding stability, the percent stabilities of all analytes were above 90% under various temperature conditions, including 4 °C and room temperature. This finding suggests that the analytes exhibit robust stability under different storage conditions, reinforcing the suitability of our method for sample storage and subsequent analysis.

By meticulously evaluating extraction recovery, matrix effect, and stability, we ensure the accuracy and reliability of our quantitative mass spectrometry approach. These assessments provide valuable insights into the performance characteristics of the method, demonstrating its effectiveness in accurately measuring the targeted analytes while minimizing potential interferences.

**Fig. 2:**
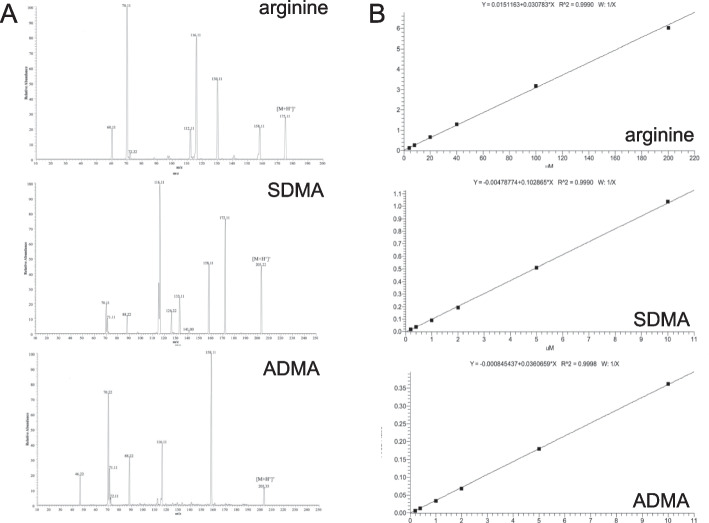
LC MS/MS Analysis of Targeted L-ARG/NO Pathway Analytes: Arginine, SDMA, and ADMA (**A**) LC MS/MS Fragmentation Spectra: The fragmentation spectra obtained through LC MS/MS analysis for the targeted analytes, namely arginine, SDMA, and ADMA, are presented. **B** Calibration Curves for the LC MS/MS Assays: The calibration curves generated for the LC MS/MS assays are shown. These curves depict the relationship between the concentration of each analyte and its corresponding peak area response. By plotting a series of known concentrations and their respective peak areas, the curves enable accurate quantification of the analytes in unknown samples based on their peak area values

In the initial stages of mass spectrometric (MS) method development, the optimization of selected reaction monitoring (SRM) transitions was carried out for the targeted analytes of interest. This involved individually infusing corresponding standards and internal standards (ISs) into the MS system. The MS/MS fragmentation spectra depicting this optimization process are presented in Fig. 2 (A: L-ARG, B: SDMA, C: ADMA).

During optimization, the signal counts obtained from different product ions were compared. The two most intense product ions were selected as quantifier and qualifier ions, respectively. Their optimal precursor m/z, product m/z, collision energy (CE), and radio frequency (RF) lens values were determined based on the prior optimization results. Additionally, considering the structural similarity between ADMA and SDMA, we conducted a parallel comparison of their fragmentation profiles. We identified signature fragments at 46.17 and 172.13 m/z for ADMA and SDMA, respectively, while other abundant fragments were commonly present in the fragmentation spectra of both configurational isomers. Consequently, the fragments at 46.17 and 172.13 m/z were chosen as quantifier ions for ADMA and SDMA, respectively. Since there were no secondary signature fragments and the quantifier ions demonstrated robustness in previous studies, qualifier ions were not selected for both ADMA and SDMA.

Subsequently, the cycle time was optimized based on the chromatographic peak widths of individual analytes and the total number of transitions. The candidate transitions were then evaluated against blank and pooled serum samples to assess their sensitivities and specificities, both with and without potential matrix interferences. The selected transitions exhibited satisfactory performance using the optimized cycle time.

The linearity of the calibration curve, determined by the coefficient of determination (r2) from linear regression fitting, was assessed for each analyte using a 6-level calibration. Figure 2B displays the calibration curves, demonstrating an r2 greater than 0.99 for L-ARG, ADMA, and SDMA. This confirms the excellent linearity of the calibration curves for these analytes.

Through the process of SRM transition optimization and calibration curve determination, we have successfully established robust and linear MS assays for the targeted analytes L-ARG, ADMA, and SDMA. These optimized methods provide reliable and accurate quantification of the analytes of interest.

## Results

### Comprehensive analysis of tissue gene expression in ovarian cancer: insights from diverse datasets and functional modules

**Fig. 3:**
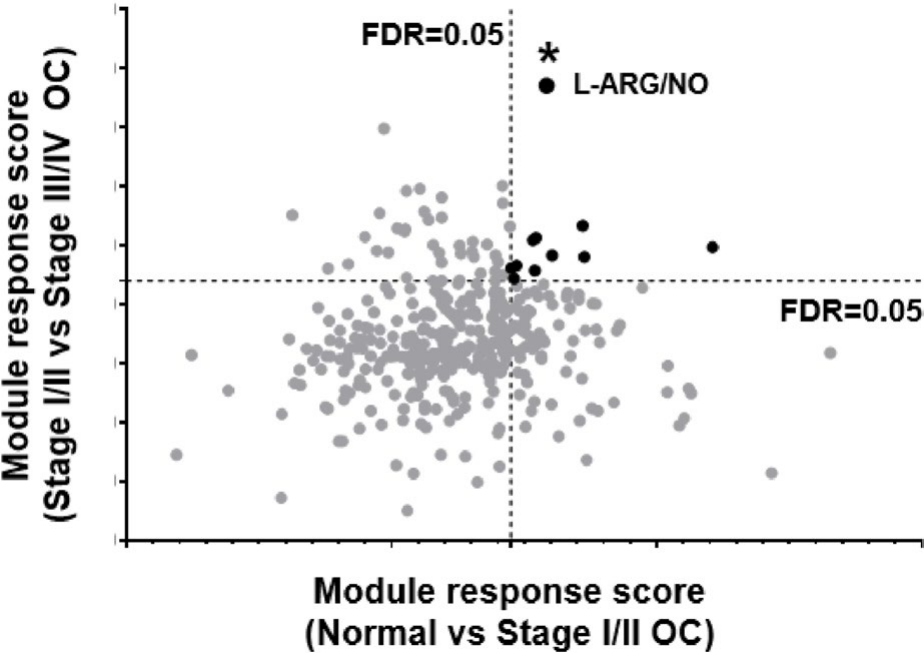
Tissue gene expression functional module analysis. Panel A. False discovery analysis (FDR) revealed significant functional modules differentiating normal from stage I/II or I/II from III/IV ovarian cancer. The horizontal axis: module response score comparing normal versus stage I/II cancer. The vertical axis: module response score comparing stage I/II versus III/IV cancer. L-ARG/NO: L-arginine/nitric oxide pathway

In this study, we utilized tissue gene expression datasets from GEPIA2 [12] and GENT2 [13] databases, encompassing normal, benign, and early/late ovarian cancer samples. A total of 18 gene expression profiling datasets of ovarian cancer tissues were compiled, comprising 71 normal ovary, 8 benign, 280 early stage (stage I and II), and 1016 late stage (stage III and IV) ovarian cancer tissue samples. Through meticulous analysis, we identified 11 significant (FDR < 0.05) functional modules that distinguish normal from stage I/II or stage I/II from III/IV ovarian cancer (Fig. 3). These functional modules encompass key biological processes such as sphingolipid metabolism, histidine metabolism, phenylalanine, tyrosine and tryptophan biosynthesis, ribosome biogenesis in eukaryotes, base excision repair, homologous recombination, non-homologous end-joining, signaling pathways regulating pluripotency of stem cells, DNA replication, DNA repair and recombination, and L-ARG/NO pathway.

### The significance of the L-ARG/NO pathway in ovarian cancer: a comprehensive analysis

Our analysis of tissue gene expression datasets has revealed intriguing findings regarding the L-ARG/NO pathway in ovarian cancer. Among the 11 most discriminant functional modules identified during the false discovery functional module analyses (Supplementary data 1), the L-ARG/NO pathway stood out prominently. This observation was made when comparing normal tissue to early-stage ovarian cancer, as well as when comparing early-stage to late-stage ovarian cancer (Fig. 3).

To further investigate the relationship of the L-ARG/NO pathway with other functional modules, we performed a module minimum spanning tree analysis (Supplementary data 2 Fig. 1). In the comparison of normal tissue versus stage I/II ovarian cancer, the L-ARG/NO pathway directly clustered with the "DNA replication" module. In the comparison of stage I/II versus stage III/IV ovarian cancer, the L-ARG/NO pathway indirectly connected to the "DNA replication" module through the "Base excision repair" module.

**Fig. 4:**
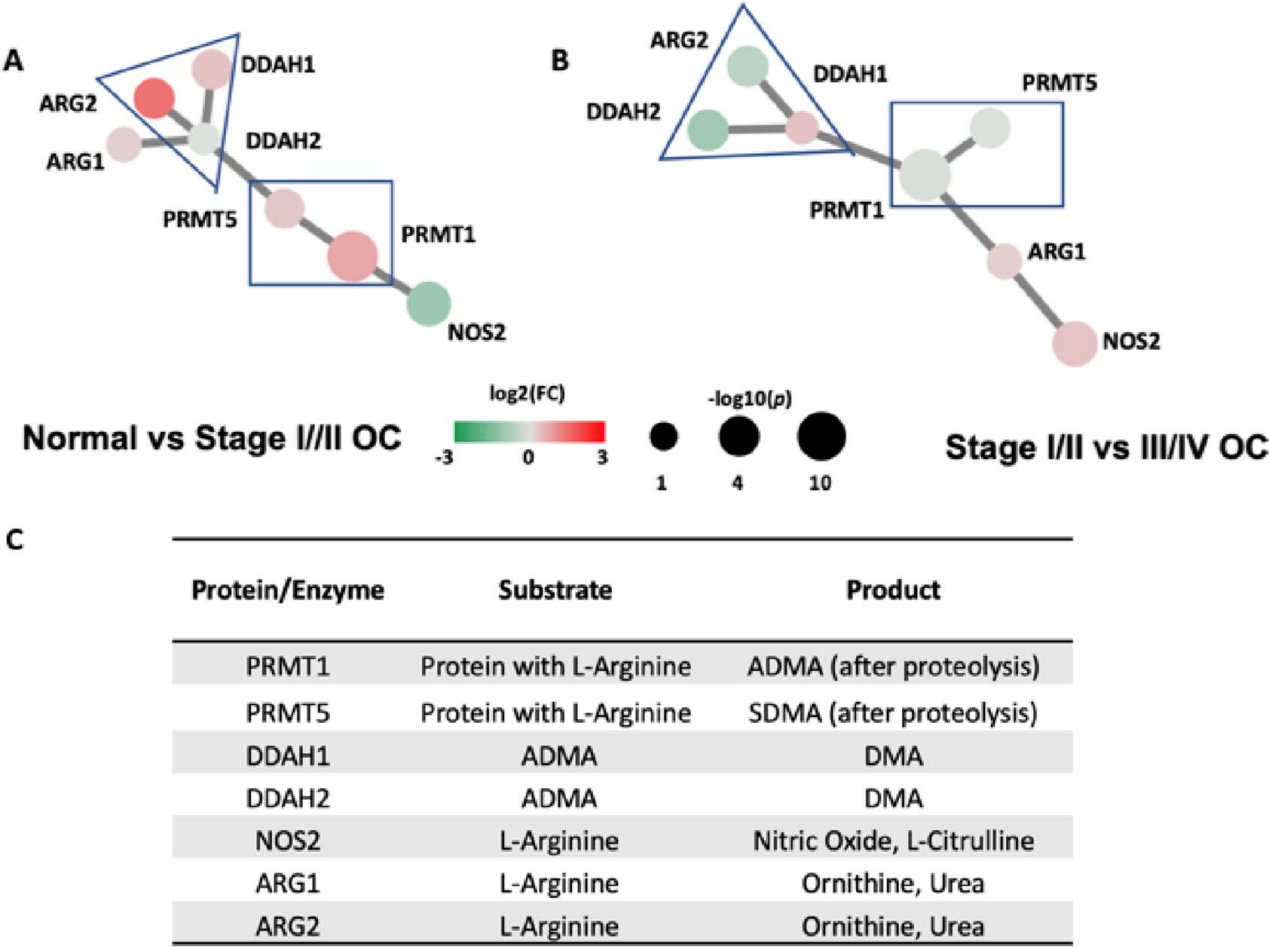
L-arginine/nitric oxide module gene expression pattern organized in a correlation minimum spanning tree. L-ARG/NO component genes include protein methyltransferases (PRMT1 and PRMT5), nitric oxide synthase (NOS), arginases (ARG1 and ARG2), dimethylarginine dimethyl-aminohydrolases (DDAH1 and DDAH2). The edge length between the discriminant nodes is proportional to the univariate correlation between the two genes within the module. **A** Normal vs Stage I/II OC, **B** Stage I/II vs III/IV OC gene expression comparative analysis, the size and color of the discriminant nodes are proportional to the -log (*p* value) and fold of change respectively; **C** L-ARG/NO pathway key enzymes, enzyme substrates, and enzyme products

**Fig. 5:**
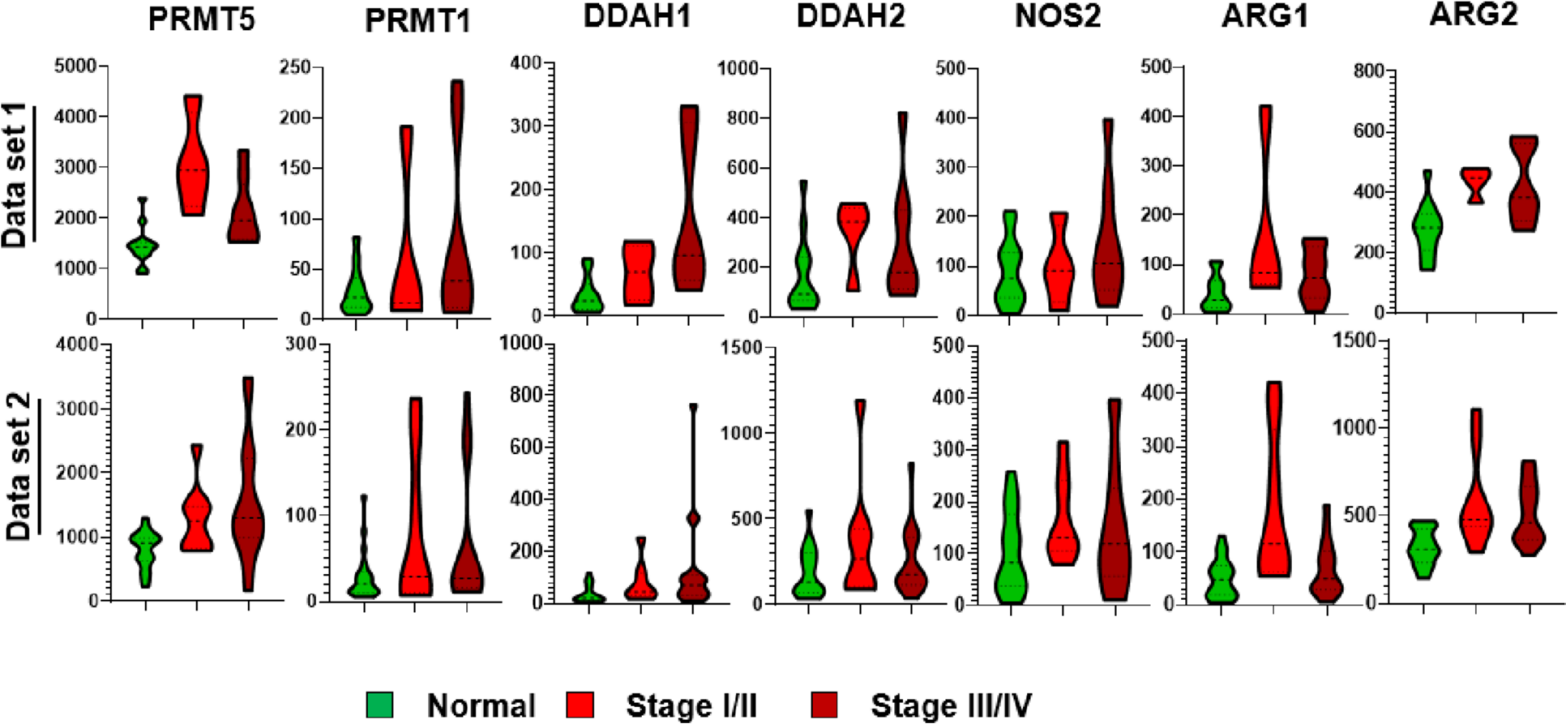
L-ARG/NO pathway and its key pathway gene expression differentiation in ovarian cancer

The L-ARG/NO pathway comprises several genes involved in key biological processes. These include protein methyltransferases (PRMT1 and PRMT5), nitric oxide synthase (NOS), arginases (ARG1 and ARG2), and dimethylarginine dimethylaminohydrolases (DDAH1 and DDAH2). Through the minimum spanning tree analysis (Fig. 4), we observed unique clustering patterns and gene expression profiles of these seven component genes. Notably, gene clusters of PRMT1/PRMT5 and DDAH1/DDAH2/ARG2 persisted, while other gene tree relationships and expression significances differed between the two comparisons: normal tissue versus stage I/II ovarian cancer (Fig. 5A) and stage I/II versus stage III/IV ovarian cancer (Fig. 5B).

To further validate our findings, we examined the expression patterns of the component genes using two individual gene expression datasets: GSE14407 [14] and GSE38666 [15, 16]. These datasets included normal tissue samples as well as stage I/II ovarian cancer tissues. As depicted in Fig. 5, PRMT5, PRMT1, DDAH1, DDAH2, NOS2, ARG1, and ARG2 were all found to be up-regulated in the stage I/II or stage III/IV ovarian cancer tissues compared to normal tissues.

To conclude, our comprehensive analysis highlights the significance of the L-ARG/NO pathway in ovarian cancer.

### L-Arginine/nitric oxide pathway and associated substrate/product metabolites

Summarized in Fig. 4, the seven L-ARG/NO module gene members code enzymes involved in the methyl group transfer, protein degradation, and arginine metabolism to obtain several biologically active substances including NO, ornithine, asymmetric (ADMA) and symmetric (SDMA) dimethylarginines, and dimethylamines (DMA).

Dimethylarginines are the degradation products of proteins, methylated by protein methyltransferases (PRMTs). PRMT1 is responsible for the most of ADMA yield and PRMT5 is responsible for the release of SDMA. NO synthases and arginases metabolize arginine, a semi-essential amino acid, to obtain NO and ornithine. Our gene expression analysis (Figs. 4A/B and 5) showed significantly differentiated PRMT5 and ARG1 expression in ovarian cancers with more than two-fold of increase and *p* value < 0.001.

### Quantitative LC–MS/MS assays reveal altered metabolite levels in ovarian cancer plasma

To investigate the metabolic changes associated with ovarian cancer (OC), we employed LC–MS/MS-based quantitation assays to measure the levels of asymmetric dimethylarginine (ADMA), symmetric dimethylarginine (SDMA), and arginine in plasma samples obtained from an OC patient cohort (Table 1, Supplementary data 2 Table 1).

**Fig. 6:**
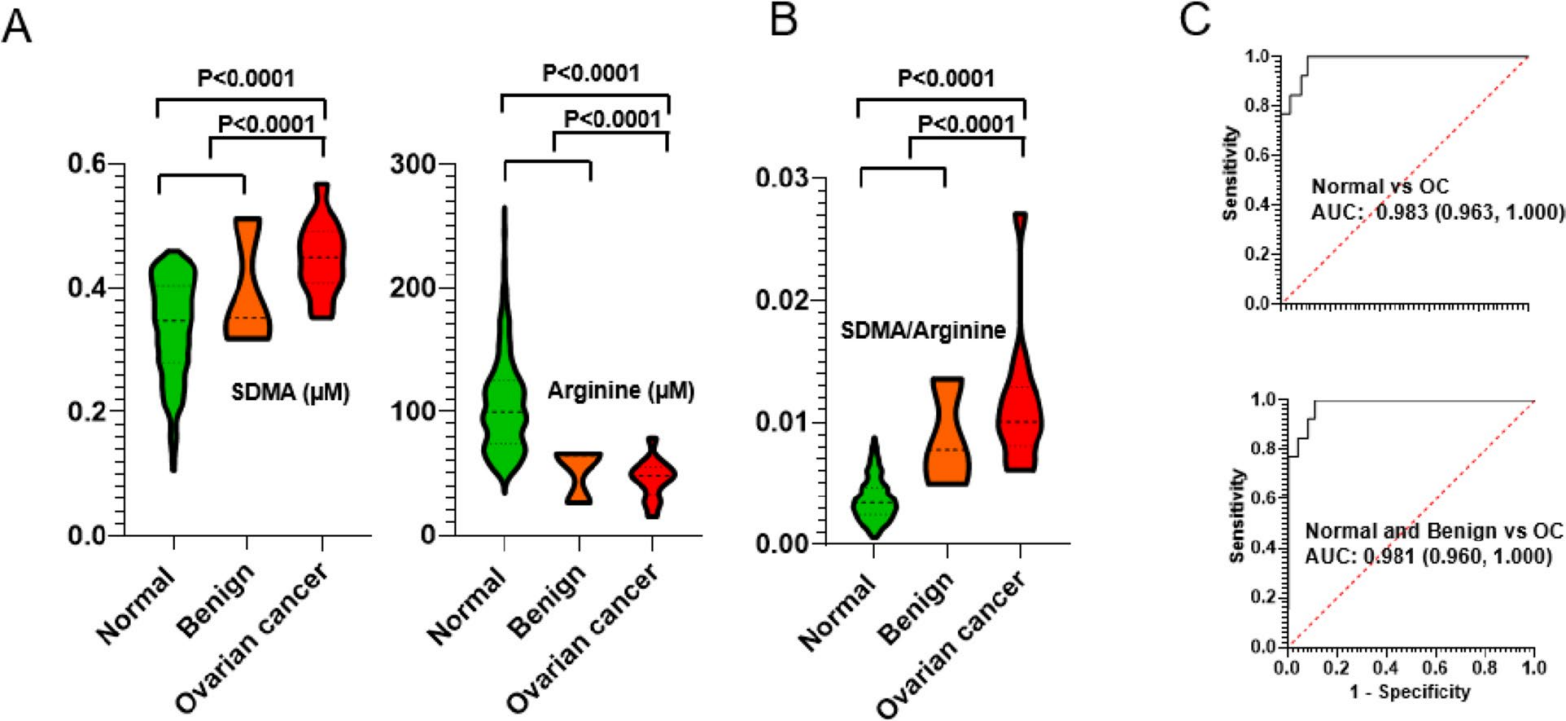
Quantitative analysis of the targeted L-ARG/NO pathway analytes: arginine, SDMA and ADMA. **A** Blood analyte abundance in normal, stage I, III, and IV samples. **B** SDMA/ARG ratio as a biomarker to classify different stage cancers from the normal controls

Our findings revealed significant differences in the plasma levels of SDMA and arginine between OC patients and individuals with normal or benign tumor conditions. As depicted in Fig. 6A, the plasma level of SDMA was significantly elevated (*p* value < 0.0001) in OC patients compared to both normal and benign tumor samples. Conversely, the level of arginine in OC sera was significantly decreased (*p* value < 0.0001). Interestingly, as key metabolites in the L-ARG/NO pathway, SDMA and arginine exhibited opposite changes in plasma levels among OC patients, suggesting that their ratio could potentially serve as a biomarker for OC assessment.

To evaluate the diagnostic performance of the SDMA-to-arginine ratio, we compared it to the individual metabolites. As shown in Fig. 6B/C, the statistical differentiation achieved by the ratio outperformed that of SDMA or arginine alone in distinguishing OC samples from normal samples (ROC AUC: 0.983) or from combined normal and benign tumor samples (ROC AUC: 0.981). These results indicate that the SDMA-to-arginine ratio holds promise as a potential biomarker for OC detection and differentiation.

## Discussion

We have described the identification of a biomarker ratio, specifically the L-ARG/NO module metabolite biomarker ratio, which promises the enabling of the liquid biopsy assessment of ovarian cancer. Our gene expression module analysis with a large cohort of ovarian cancer tissue biopsy samples led to the identification of functional modules that significantly differentiate normal tissue from stage I/II or stage I/II from III/IV ovarian cancer. Among the top 11 significant modules associated with ovarian cancer, the L-ARG/NO module stood out. Through the characterization of the reprogrammed metabolic L-ARG/NO pathway in ovarian cancer tissue, we hypothesized that the pathway metabolite SDMA and arginine could serve as potential liquid biopsy biomarkers for assessing ovarian cancer. To test this hypothesis, we developed a sensitive and specific quantitative mass spectrometric assay that allows for the parallel analysis of circulating SDMA and arginine. Analyzing a pilot cohort of blood samples from ovarian cancer patients, we demonstrated the potential diagnostic utility of the SDMA-to-arginine ratio as a liquid biopsy biomarker for ovarian cancer assessment.

One of the main strengths of our study lies in the use of a quantitative agnostic framework to compare different stages of ovarian cancer. We integrated tumor tissue mRNA expression data and biological functional module networks to identify 11 significant modules that differentiate normal tissue from early-stage ovarian cancer or early-stage from late-stage ovarian cancer. These modules consist of genes known to play roles in cancer biology or specifically in ovarian cancer pathophysiology. We visually represented the interactions of these 11 differentiating functional modules in a correlation minimum spanning tree. By closely examining these modules, we found that "DNA replication" and "DNA repair and recombination" modules were central in connecting other functional modules in both normal versus early and early versus late comparisons. Hallmarks of cancer cells include uncontrolled cell proliferation, genome instability, and mutations. Therefore, targeting DNA repair and replication stress has been proposed in the OC treatment [17, 18]. Approximately half of high-grade serous epithelial ovarian cancers incur alterations in genes of homologous recombination (BRCA1, BRCA2, RAD51C, Fanconi anemia genes), and the rest incur alterations in other DNA repair pathways at high frequencies. These previous findings are in line with our identification of the following significant functional modules underlying OC: DNA replication, DNA repair and recombination, the base excision repair, homologous recombination, and non-homologous end-joining. Cancer stem cells (CSCs) were found to correlate to ovarian cancer, locating in patient ascites [19], the ovarian surface [20] and fallopian tube epithelium [21]. Our identification of the OC significant module, “signaling pathways regulating pluripotency of stem cells”, is consistent with the notion that CSCs potentially underlie the biology of ovarian cancer growth and frequent relapse [22, 23]. Sphingolipids are multifaceted mediators in ovarian cancer [24], and the sphingosine kinase 1-sphingosine 1-phosphate receptor 1 axis is altered in ovarian cancer in multiple ways and therefore represents an attractive therapeutic target. Recent targeted metabolomics analyses [25, 26] concerning the role of amino acids in OC identified histidine metabolism factors as potential new OC biomarkers. Large-scale profiling of metabolic dysregulation in ovarian cancer found aromatic amino acids (phenylalanine, tyrosine and tryptophan) alterations, and such metabolic reprogramming may be important to achieve metabolic harmony in ovarian cancer [27, 28]. Signaling pathways upstream of the module ribosome biogenesis [29], including the PI3K/AKT/mTORC1 and RAS/MAPK signaling pathways and the c-MYC proto-oncogene “super” growth regulatory network, are aberrantly regulated and activated in ovarian cancer. Thus, developing therapeutics to target ribosome biogenesis has emerged as a novel approach against ovarian cancer.

The L-ARG/NO pathway emerged as one of the top functional modules associated with ovarian cancer in our study. Nitric oxide synthase 2 (NOS2), a biomarker of early cancer development, cancer progression, and patient survival, has been implicated in breast cancer, brain metastases, and ovarian cancer. Protein arginine methyltransferases (PRMTs), particularly PRMT5 and PRMT1 identified in our study, are overexpressed in various cancers, including ovarian, lung, multiple myeloma, and breast tumors. Clinical trials are underway to evaluate the effectiveness of PRMT5 inhibitors in treating different types of cancer. While no clinical trial of a PRMT5 inhibitor for ovarian cancer has been initiated, several studies have demonstrated the inhibition of ovarian cancer cell growth and induction of apoptosis with PRMT5 inhibition or siRNA knockdown.

The L-ARG/NO pathway metabolites have been previously identified as cancer biomarkers in various metabolic profiling studies. For instance, lower levels of serum arginine and citrulline were found in colorectal cancer samples compared to control samples. The metabolic differentiation of arginine and citrulline in colorectal cancer tissues was associated with the overexpression of the arginine transporter gene CAT-1. Therefore, systemically deregulated metabolites of the L-ARG/NO pathway hold promise as predictors of adverse clinical events after curative tumor resection.

The L-arginine/nitric oxide (L-ARG/NO) pathway plays a critical role in ovarian cancer progression. This pathway involves the metabolism of L-arginine, an amino acid, by the enzyme nitric oxide synthase (NOS) to produce nitric oxide (NO). The dysregulated L-ARG/NO pathway led to alterations in the levels of L-ARG and NO in the tumor microenvironment, contributing to ovarian cancer progression and aggressiveness. Nitric oxide is a signaling molecule that regulates various cellular processes, including cell growth, angiogenesis (formation of new blood vessels), promotion of the remodeling of the extracellular matrix and facilitation of the tumor cell invasion, immune responses, and its association with resistance to chemotherapy.

Metabolic reprogramming is a hallmark of malignancy [30–33], and reprogrammed metabolic activities can be exploited to diagnose, monitor, and treat cancer. Based on our gene expression analyses of the L-ARG/NO module and up-regulation of PRMT1/5/ARG1/2, we hypothesized that the pathogenesis and pathophysiology of ovarian cancer might be associated with systemic and circulating changes in SDMA and L-ARG metabolites. Particularly, we proposed that the up-regulated metabolic ratio of SDMA-to-L-ARG could serve as a biomarker for ovarian cancer assessment. Our findings of these metabolite biomarkers, if validated in ovarian cancer cohorts, could provide valuable insights into the cause-specific mechanisms of cancer pathogenesis, progression and metastasis, response to therapy, and overall function.

While our functional module discovery analysis effectively identified differentiated pathways and hypothesized associated metabolites as potential biomarkers, the clinical translation and field application of these findings rely on absolute quantitative liquid biopsy data [34, 35]. To this end, we developed MRM/SID-MS assays for SDMA and arginine following FDA Bioanalytical Method Validation Guidance for Industry. This approach allows for the absolute quantification of analyte abundance in blood. Our blood cohort analysis demonstrated that the SDMA-to-arginine ratio biomarker promises to have the potential to effectively differentiate control samples from ovarian cancer samples.

Our study has several primary limitations that need to be addressed. Firstly, the small sample size of the pilot cohort used for blood test analysis and the limited sample sizes within each cancer stage subgroup can compromise the generalizability, accuracy, and clinical relevance of our conclusions. It is important to note that due to the low prevalence of ovarian cancer in the general population, an effective screening strategy must exhibit high sensitivity for early-stage disease (> 75%) and very high specificity (99.6%) to minimize unnecessary exploratory operations. This requirement is particularly crucial for postmenopausal women over 50 years of age, who are at a significantly higher risk than younger women. To validate the diagnostic utility of our biomarkers derived from the small cohort, large cohorts are necessary to ensure high sensitivity and specificity. Future follow-up studies with adequately powered cohorts should be planned to prospectively evaluate and validate the clinical utility of the SDMA-to-ARG ratio in early ovarian cancer detection, triage of pelvic mass patients for prophylactic intervention, and liquid biopsy surveillance of minimal residual disease for relapse management. Secondly, we need to consider potential limitations related to our bioinformatic or computational approaches. The choice of bioinformatic or computational algorithms employed for data analysis can significantly influence the results. It is important to acknowledge that data preprocessing steps, such as normalization and imputation, can introduce biases or distortions in the analysis. Moreover, complex bioinformatic models may be prone to overfitting the data, especially when dealing with small sample sizes. For example, limitations of our MST analysis include parameter selection, sensitivity to data preprocessing, and interpretation challenges. Therefore, It is important for the future follow up studies to consider alternative methods as well, such as hierarchical clustering, spectral clustering, k-means clustering, as well as community detection approaches like Louvain or modularity-based algorithms. Lastly, our study relies on quantitative metabolomic analysis, which necessitates the use of liquid chromatography-mass spectrometry (LC–MS) or LC–MS/MS. However, the implementation of LC–MS/MS as a diagnostic platform in the clinical setting poses numerous challenges. While LC–MS/MS offers high sensitivity, specificity, and the ability to quantify multiple analytes simultaneously, there are practical considerations that make it difficult for routine clinical diagnostics. These considerations include the requirement for specialized instrumentation and expertise in mass spectrometry and chromatography, the need for trained personnel and quality control measures, challenges related to sample preparation and throughput, the necessity for standardization and calibration, potential interferences and matrix effects, the importance of clinical validation and adherence to regulatory requirements, as well as cost and accessibility issues. Nonetheless, ongoing advancements in technology, standardization efforts, and increased recognition of the potential clinical benefits are actively addressing these challenges and expanding the applications of LC–MS/MS in routine clinical practice.

The discovery of the biomarker ratio in our study highlights its potential as a valuable diagnostic tool for the sensitive and specific assessment of early-stage ovarian cancer. This is particularly significant considering the challenges associated with the late-stage presentation of ovarian cancer, often attributed to the disease's anatomical location and the absence of noticeable symptoms in its early stages. By identifying alterations in the L-ARG/NO pathway through our biomarker ratio, we can overcome these challenges and enable early detection of the disease.

Moreover, our biomarker ratio holds promise in indirectly detecting minimal residual disease (MRD), which is responsible for recurrent disease following curative treatment. MRD is not easily identified through standard clinical evaluations or radiological exams. By utilizing our biomarker ratio as a proxy for tumor-specific changes in the L-ARG/NO pathway, we can potentially identify the presence of MRD through a liquid biopsy approach. This comprehensive understanding of the disease pathogenesis and the assessment of the risk of relapse would provide clinicians with valuable insights, facilitating precise interventions and treatment escalation when necessary. Ultimately, these advancements could significantly improve ovarian cancer survival rates.

If our findings are validated with multi-center trial with sufficiently powered cohorts, the two blood metabolites with exceptional diagnostic performance could be translated into a simple blood test. This would enable high-throughput and highly accurate analysis, revolutionizing ovarian cancer care and diagnosis. The ease and reliability of this blood test would offer significant benefits in terms of accessibility and efficiency, facilitating ovarian cancer early detection and enhancing overall patient outcomes.

### Supplementary Information

**Additional file 1. **Gene expression correlation network analysis.

**Additional file 2: Supplementary 2 Table 1.** Gene expression datasets of ovarian cancer tissue studies.** Supplementary 2 Figure 1.** Quantitative analysis of the targeted L-ARG/NO pathway analytes: arginine and SDMA. (A) Blood analyte abundance in normal, stage I, III, and IV samples. (B) SDMA/ARG ratio as a biomarker to classify different stage cancers from the normal controls. (C) ROC curve of classification.

## Data Availability

The original data supporting these findings are available at any time upon request to the corresponding author. The datasets generated during the current study are available in the official website of each database. The official website of each database is as follows: GEPIA ( http://gepia.cancer-pku.cn/index.html). GENT database (http://gent2.appex.kr/gent2/).
