# Supplementary material for: Altered expression of the L-arginine/nitric oxide pathway in ovarian cancer: metabolic biomarkers and biological implications
Source: BMC Cancer. 2023 Sep 8;23:844. doi: 10.1186/s12885-023-11192-8 (PMC10492322; doi:10.1186/s12885-023-11192-8)

Gene expression correlation network analysis

The significant functional module component genes were visualized using Cytoscape[1]. Each gene was represented by a node. Based on the spearman rank correlation matrix among component genes, the correlation structure between the genes was extracted using a Minimum Spanning Tree (MST), where the length of the edges were proportional to the correlation coefficient between the two nodes. The fold change of gene expression level (log2(FC) and *p* value (-log10(*p*)) are also showed by node color and size, respectively.

The gene correlation tree inside a functional module is extracted from whole network of 11 functional modules separately. Only the genes with $p<0.05$ are labelled.

[1] Shannon P, Markiel A, Ozier O, Baliga NS, Wang JT, Ramage D, Amin N, Schwikowski B, Ideker T. Cytoscape: a software environment for integrated models of biomolecular interaction networks. Genome Res. 2003 Nov;13(11):2498-504. doi: 10.1101/gr.1239303.

2. Non-homologous end-joining

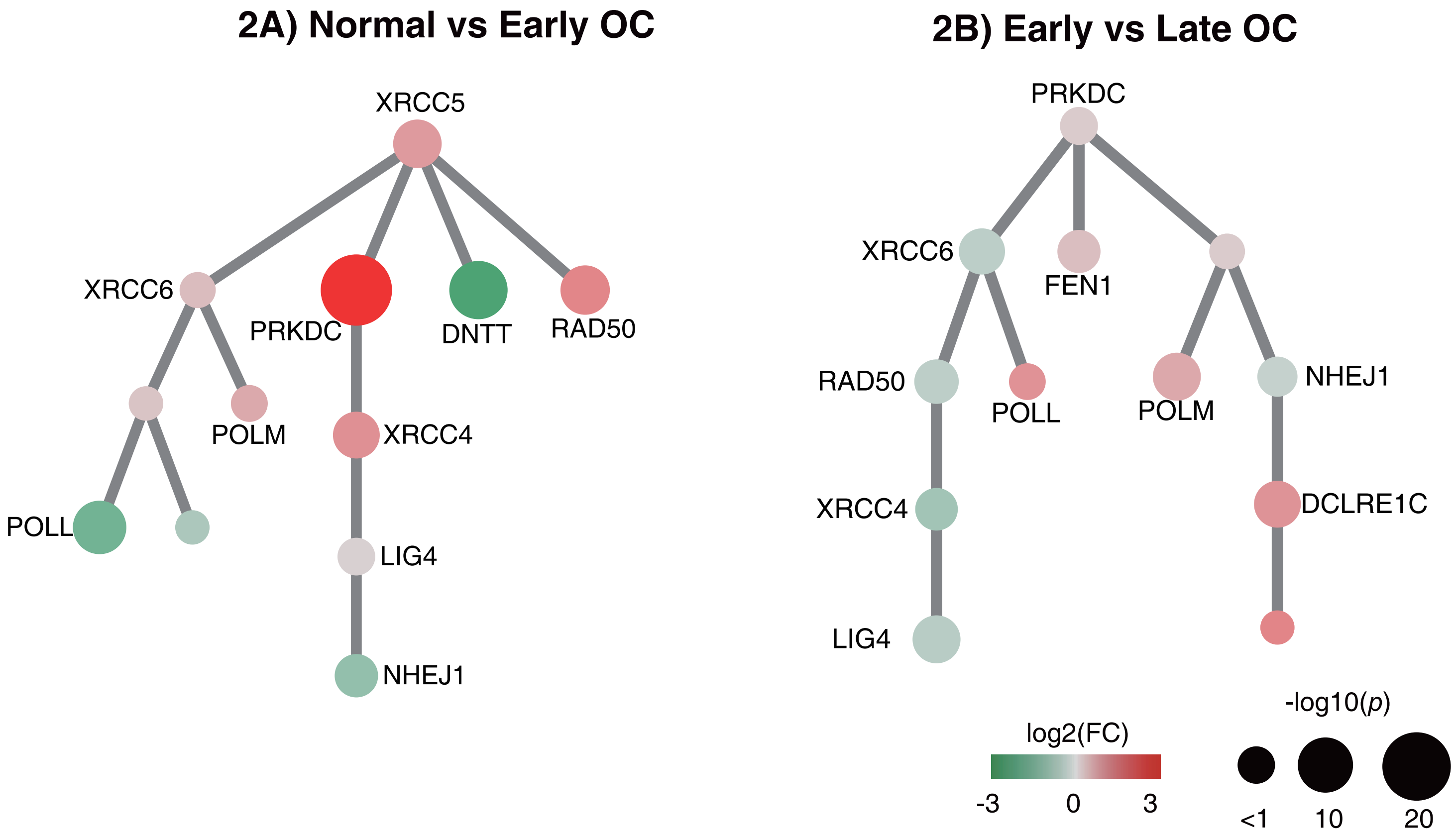

3. Phenylalanine, tyrosine and tryptophan biosynthesis

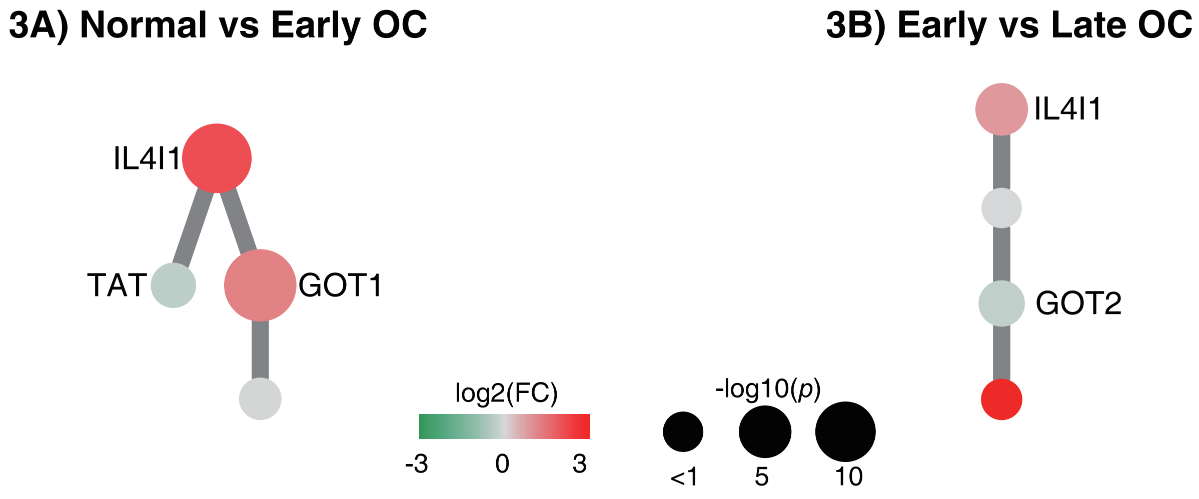

4. DNA replication

4A) Normal vs Early OC

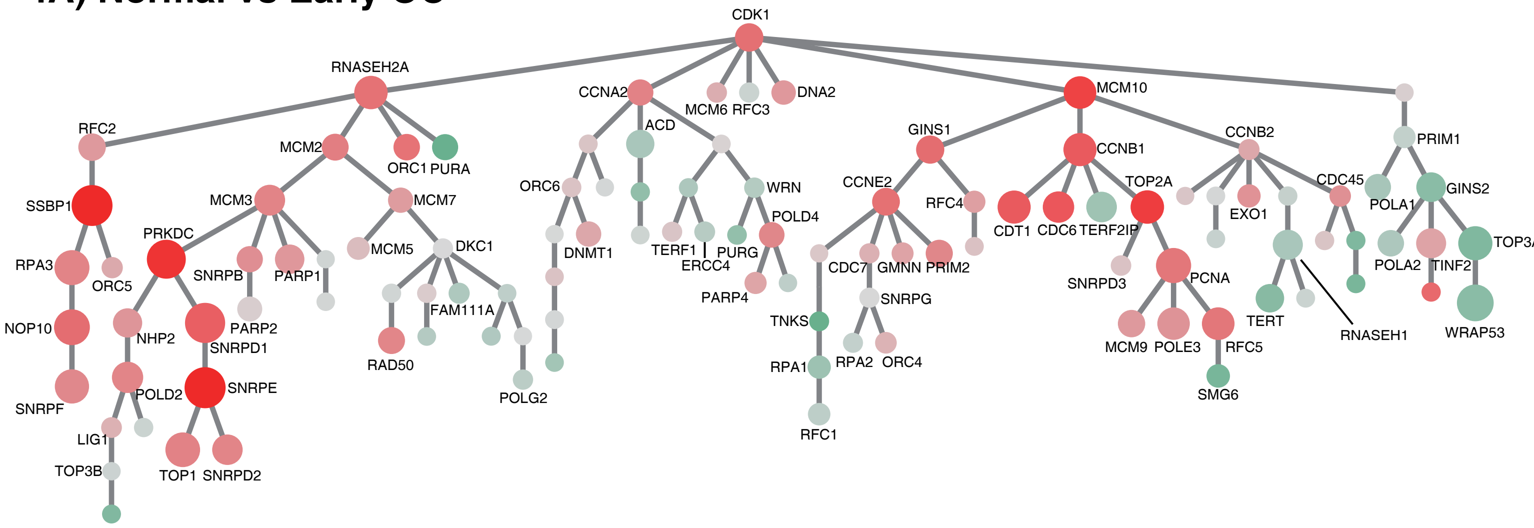

4B) Early vs Late OC

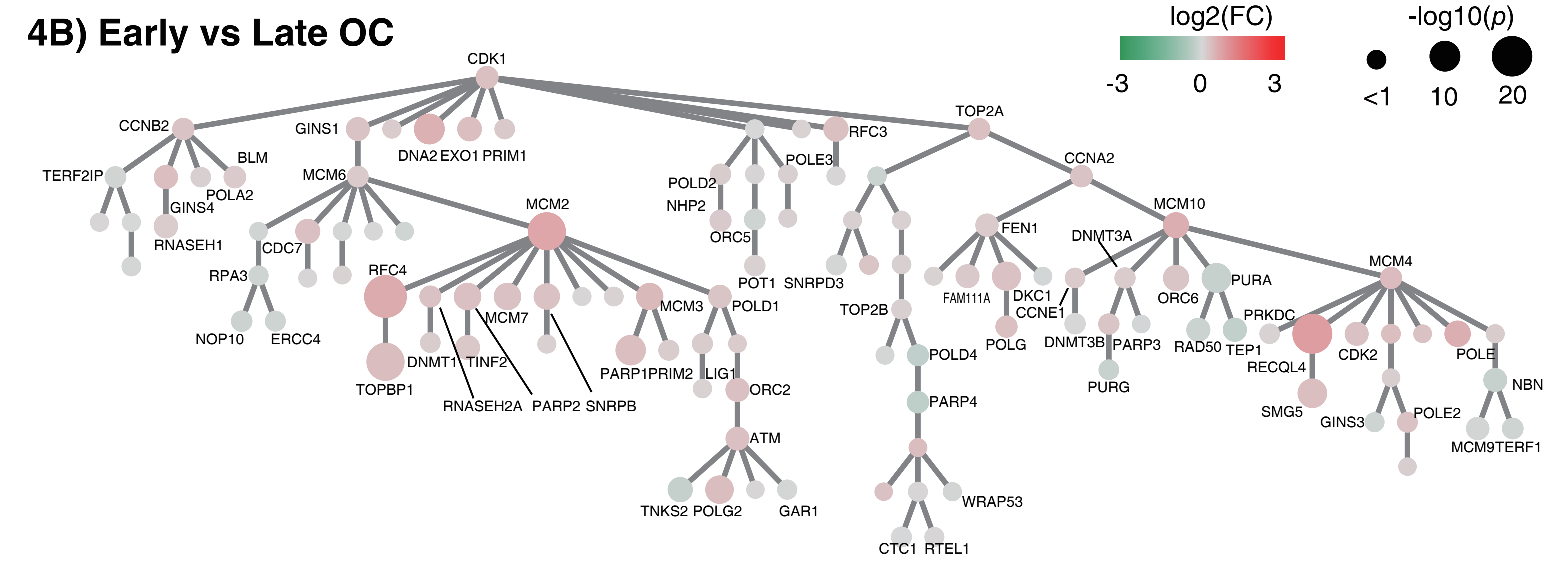

5. Base excision repair

5A) Normal vs Early OC

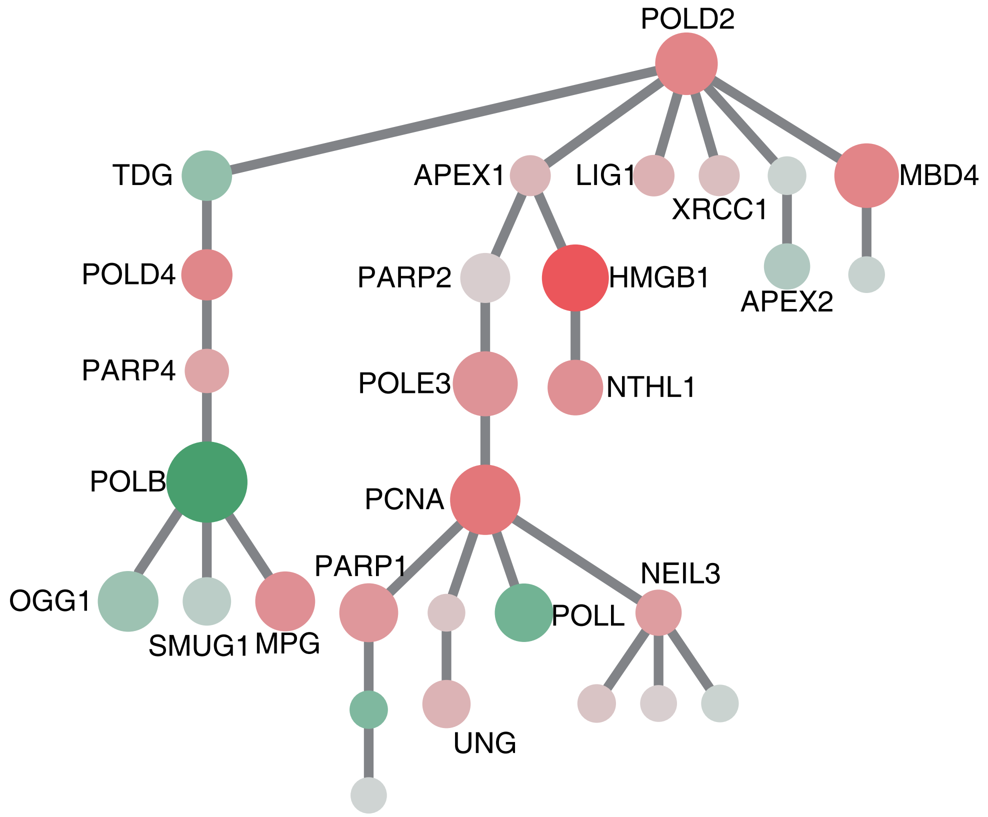

5B) Early vs Late OC

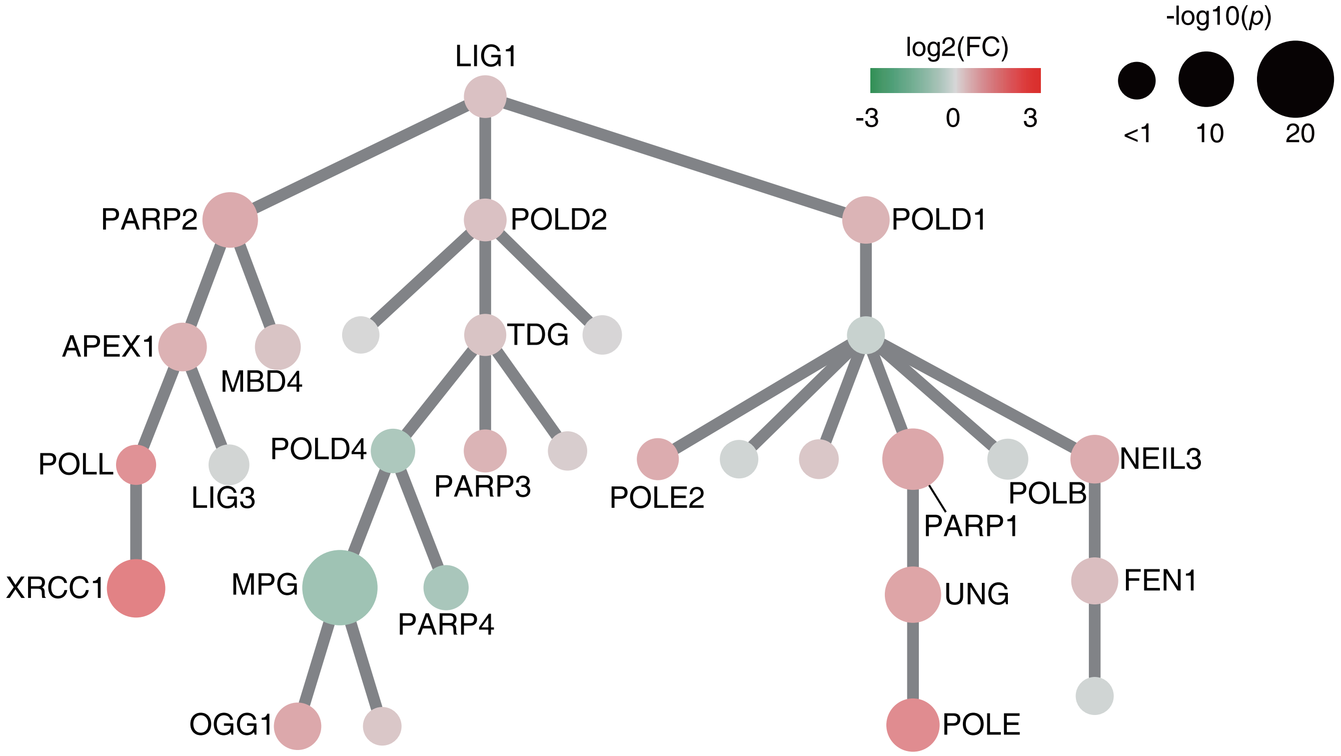

6. DNA repair and recombination

6A) Normal vs Early OC

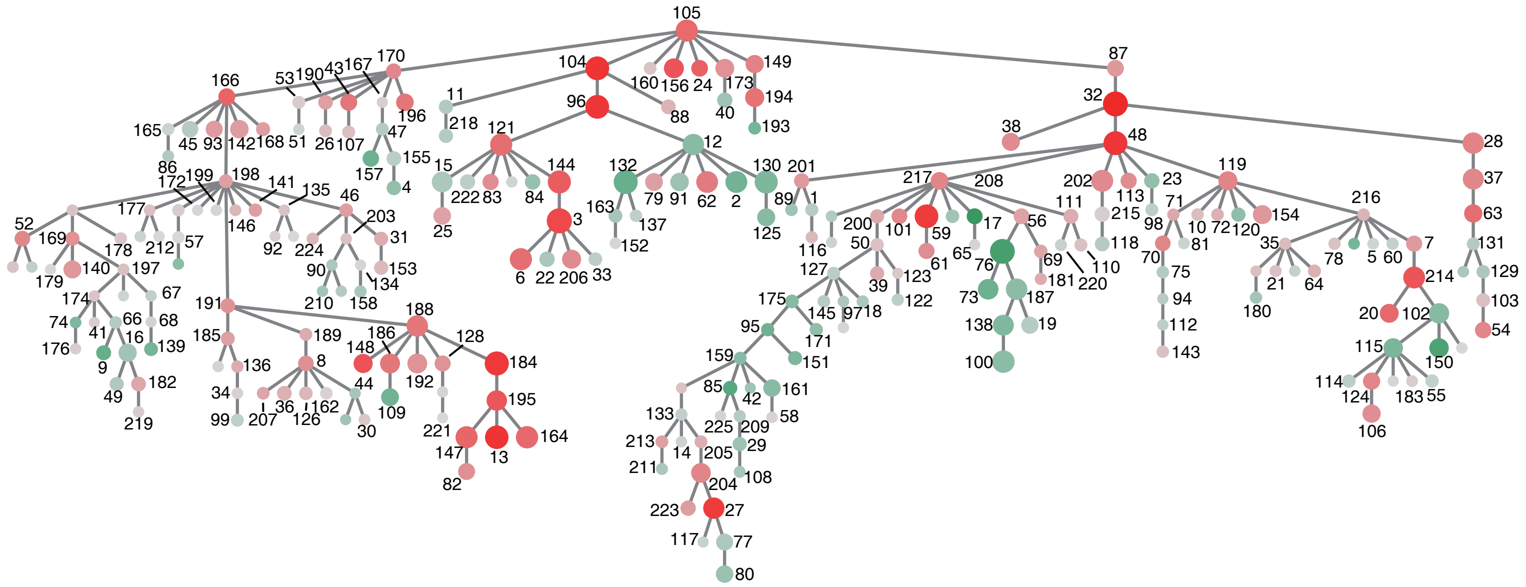

6B) Early vs Late OC

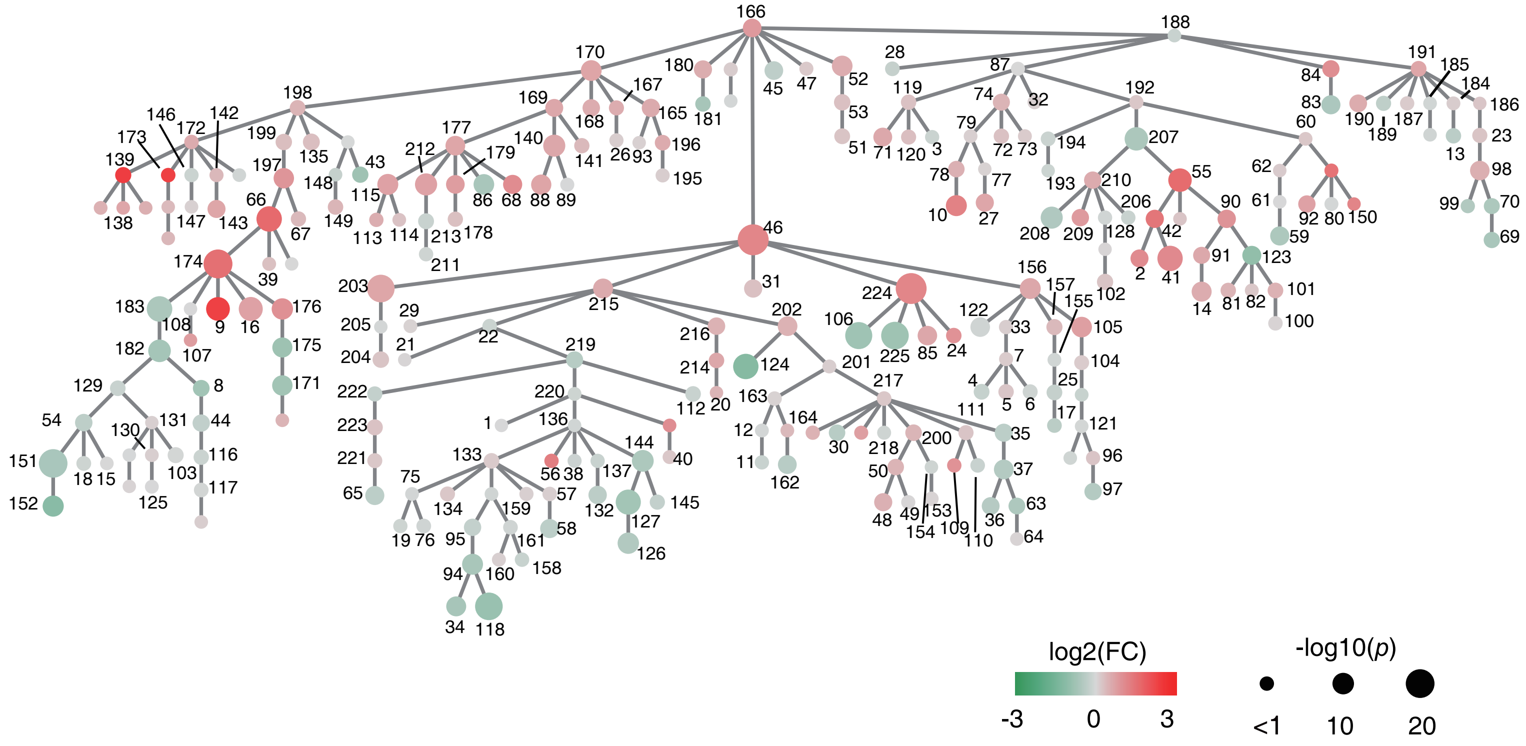

1: UBE2A
2: TDP1
3: RFC1
4: CUL3
5: YWHAB
6: POLR2F
7: EXD2
8: XRCC5
9: TDP2
10: RAD23B
11: XRCC3
12: XRCC1
13: ZCWPW1
14: PNKP
15: WEE1
16: WDR48
17: VPS4A
18: MSH4
19: UNG
20: UBE2N
21: UIMC1
22: APEX2
23: UBL4A
24: UBE2V2
25: TTI2
26: TP53
27: TOP3B
28: WRAP53
29: MUTYH
30: FANCG
31: TIMELESS
32: YWHAQ
33: TDG
34: SYCP2
35: SPO11
36: WDR76
37: RECQL5
38: SMCHD1
39: SMC6
40: RPA3
41: RPA2
42: RNF8
43: PALB2
44: POLL
45: RFC5
46: HLTF
47: RFC4
48: RAD52
49: TTI1
50: RAD54L
51: RBX1
52: RBBP8
53: RAD54L2
54: CLSPN
55: ZFYVE26
56: RAD51D
57: TOP3A
58: ERCC8
59: RAD51B
60: XPC
61: POLR2D
62: NSMCE4A
63: NEIL3
64: EXO5
65: WDR70
66: RECQL
67: PPP6R2
68: PPP4R4
69: PPP4R2
70: POLR2L
71: YWHAZ
72: NUDT15
73: NEK4
74: MLH1
75: SSBP1
76: PPP4C
77: POLR2I
78: POLR2H
79: POLR2G
80: POLR2C
81: POLR2B
82: POLI
83: POLH
84: FANCC
85: FANCA
86: POLE
87: WRN
88: POLD4
89: USP1
90: POLD3
91: LIG1
92: DDB1
93: RECQL4
94: RAD51C
95: PPP6R1
96: ATRIP
97: POLB
98: ERCC2
99: PMS2
100: POLR2K
101: PLK3
102: RFC3
103: PER2
104: RRM2
105: POLE3
106: CCNO
107: PARP4
108: POLM
109: PARP3
110: OGG1
111: NUDT1
112: NUDT18
113: SMUG1
114: NHEJ1
115: SPG11
116: NEK1
117: CETN2
118: SPIDR
119: NBN
120: REV3L
121: NAV2
122: MYBBP1A
123: YWHAE
124: PRPF19
125: DNTT
126: MUS81
127: MSH5
128: RAD50
129: MSH3
130: RFC2
131: PRKDC
132: PPP6R3
133: MSH6
134: MORF4L1
135: MGMT
136: AP5M1
137: MMS19
138: XRCC2
139: FANCE
140: CCNH
141: AP5Z1
142: LIG4
143: SAMHD1
144: LIG3
145: VPS4B
146: POLD2
147: KANK3
148: HUS1
149: PLS3
150: HSF2BP
151: RRM1
152: MPG
153: MDC1
154: HES1
155: FAN1
156: HERC2
157: UBE2B
158: RAD23A
159: NEK11
160: ERCC4
161: GTF2H5
162: TELO2
163: GTF2H4
164: YWHAH
165: RAD17
166: GTF2H3
167: GTF2H2
168: XPA
169: FANCF
170: GTF2H1
171: SMC5
172: FANCL
173: TIPIN
174: FEN1
175: DCLRE1B
176: CDC25C
177: RAD51
178: PARP1
179: CDC25A
180: EXO1
181: MLH3
182: ERCC6
183: ERCC1
184: NTHL1
185: DUT
186: POLQ
187: DMC1
188: ERCC5
189: DDB2
190: TOPBP1
191: RPA4
192: DCLRE1A
193: REV1
194: PPP4R1
195: CUL5
196: RPA1
197: CUL4B
198: HMGB1
199: CUL4A
200: CHEK2
201: RAD54B
202: RAD51AP1
203: RAD1
204: POLE2
205: BRCA2
206: BARD1
207: CHEK1
208: MNAT1
209: CDK7
210: MSH2
211: CDC5L
212: POLD1
213: CDC25B
214: RMI1
215: PCNA
216: BRIP1
217: KANK2
218: DONSON
219: BRCC3
220: BRCA1
221: FANCI
222: BLM
223: BCAS2
224: UBE2NL
225: RAD9A
226: POLR2E
227: POLR2A
228: BAG6
229: POLR2J
230: PARP2
231: BABAM1
232: ATR
233: DCLRE1C
234: ATM
235: XRCC4
236: ASCC3
237: PMS1
238: KANK1
239: APTX
240: XRCC6
241: ERCC3
242: APEX1
243: PPP6C
244: AP5S1
245: MBD4
246: ANKRD28
247: PER3
248: AEN

7. Homologous recombination

7A) Normal vs Early OC

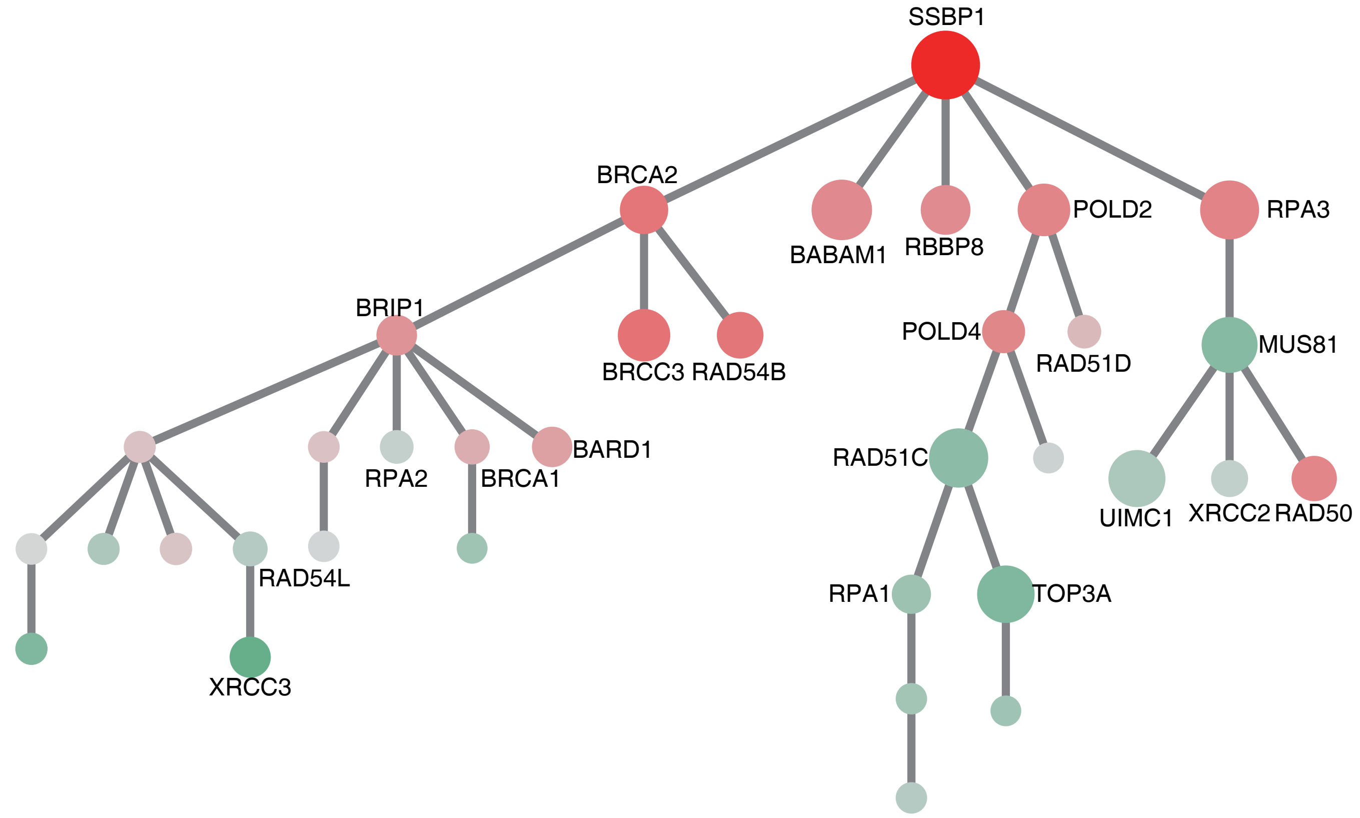

7B) Early vs Late OC

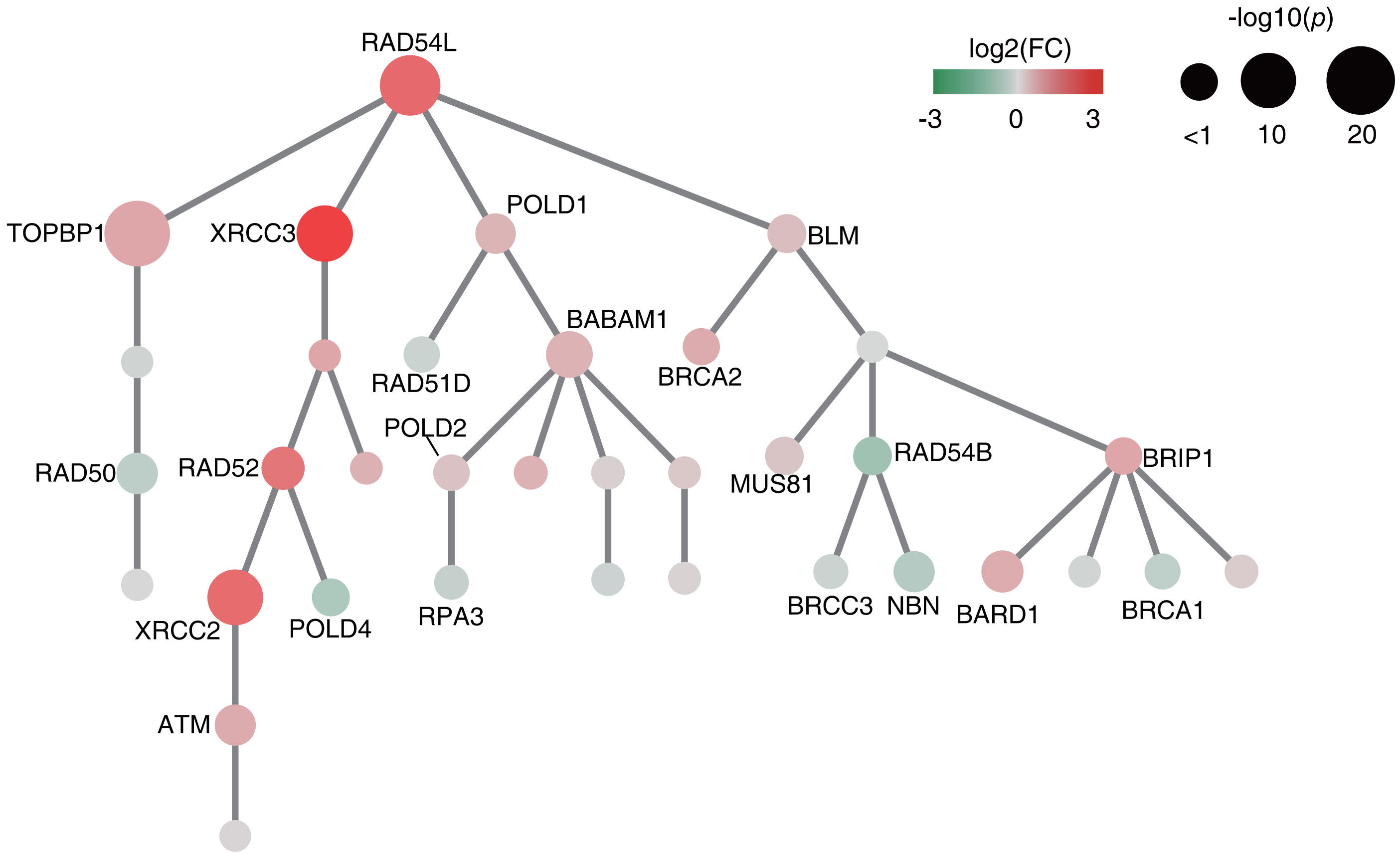

8. Ribosome biogenesis in eukaryotes

8A) Normal vs Early OC

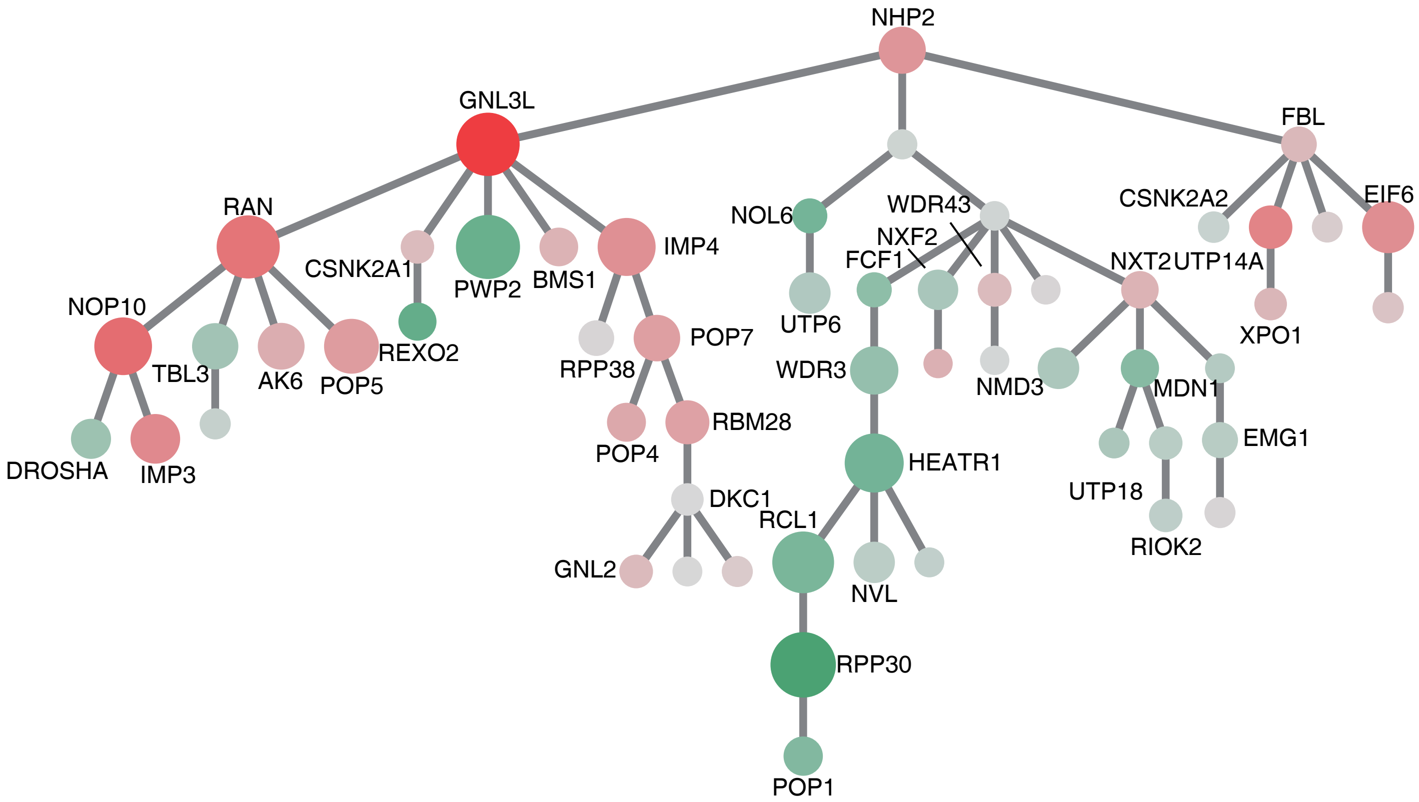

8B) Early vs Late OC

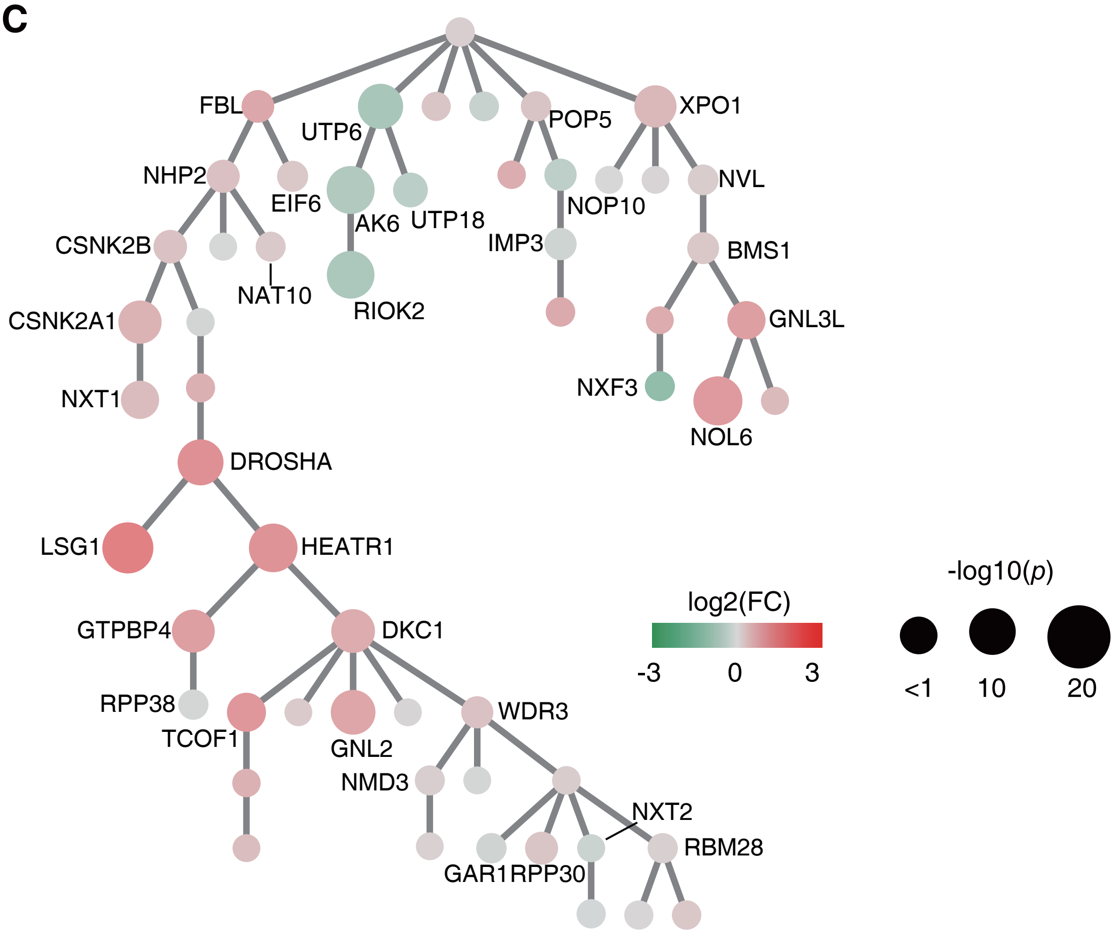

9. Sphingolipid metabolism

9A) Normal vs Early OC

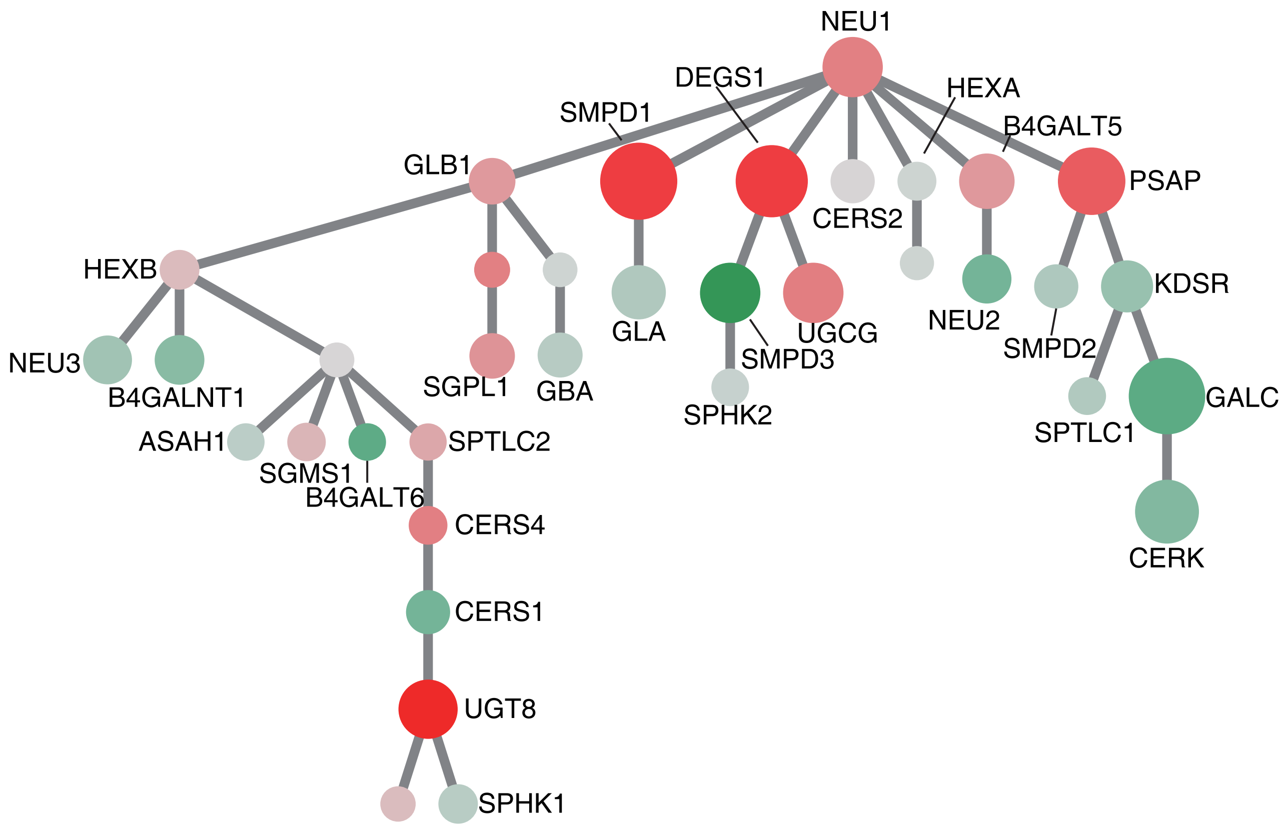

9B) Early vs Late OC

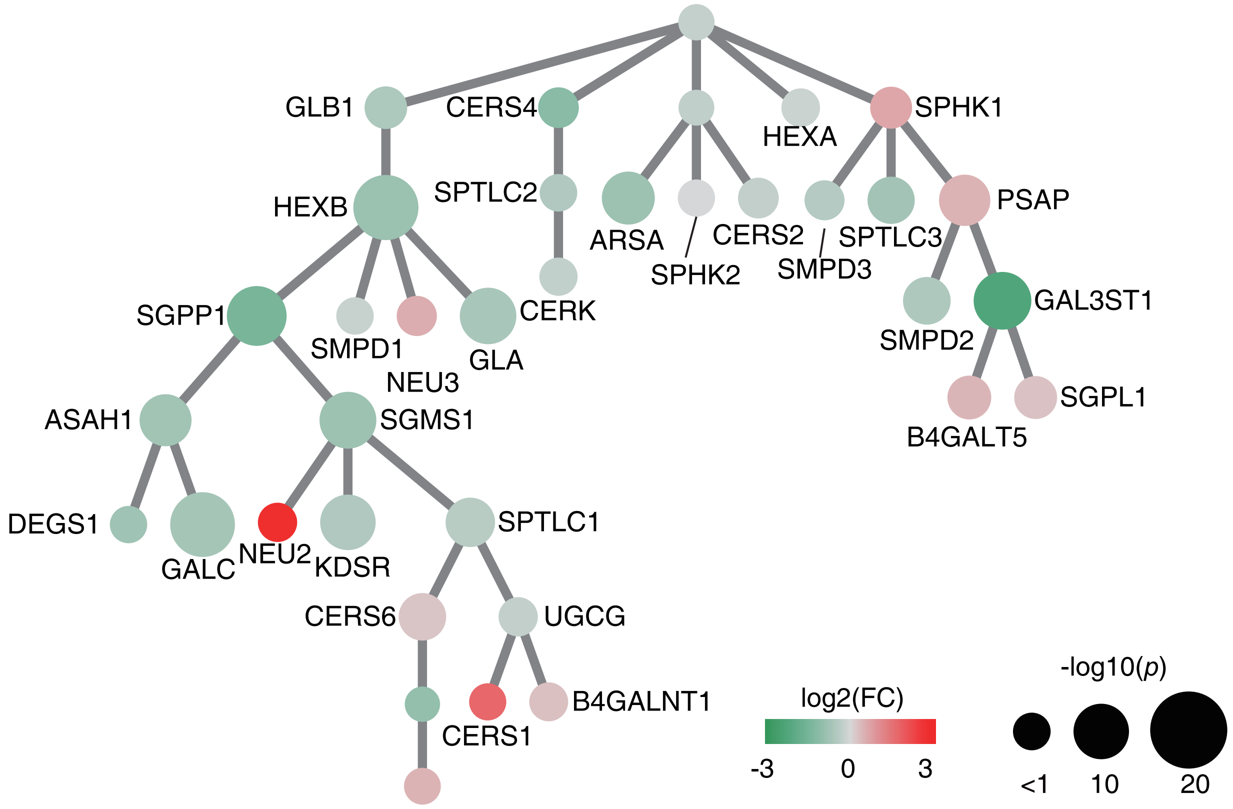

10. Stem cells pluripotency signaling

10A) Normal vs Early OC

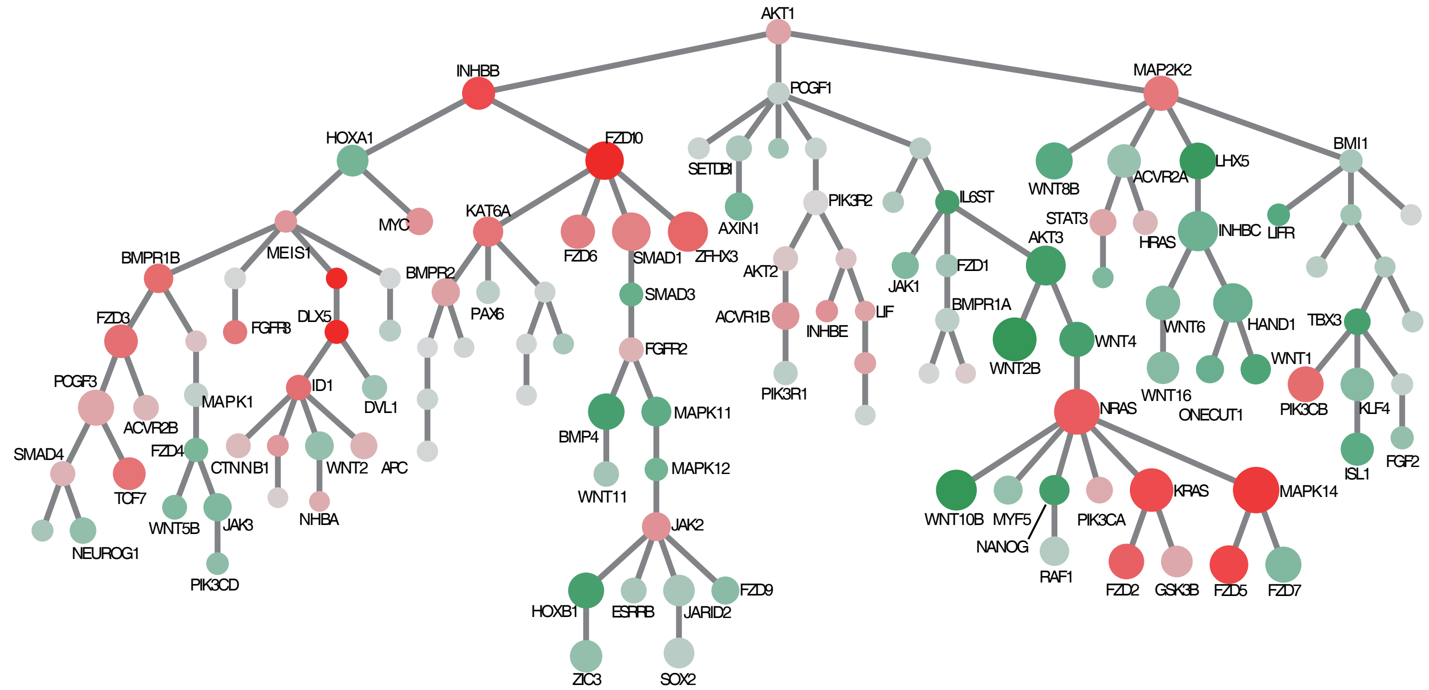

10B) Early vs Late OC

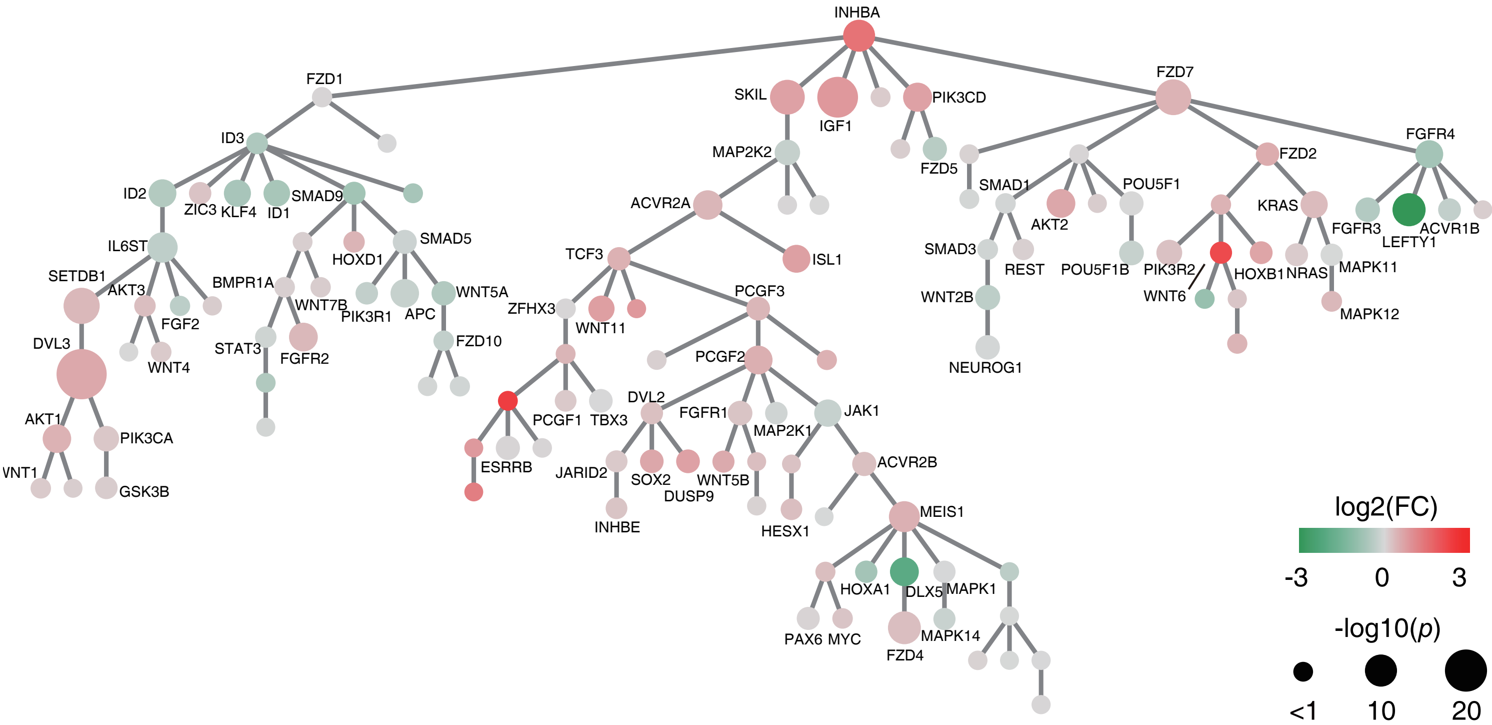

11. Histidine metabolism

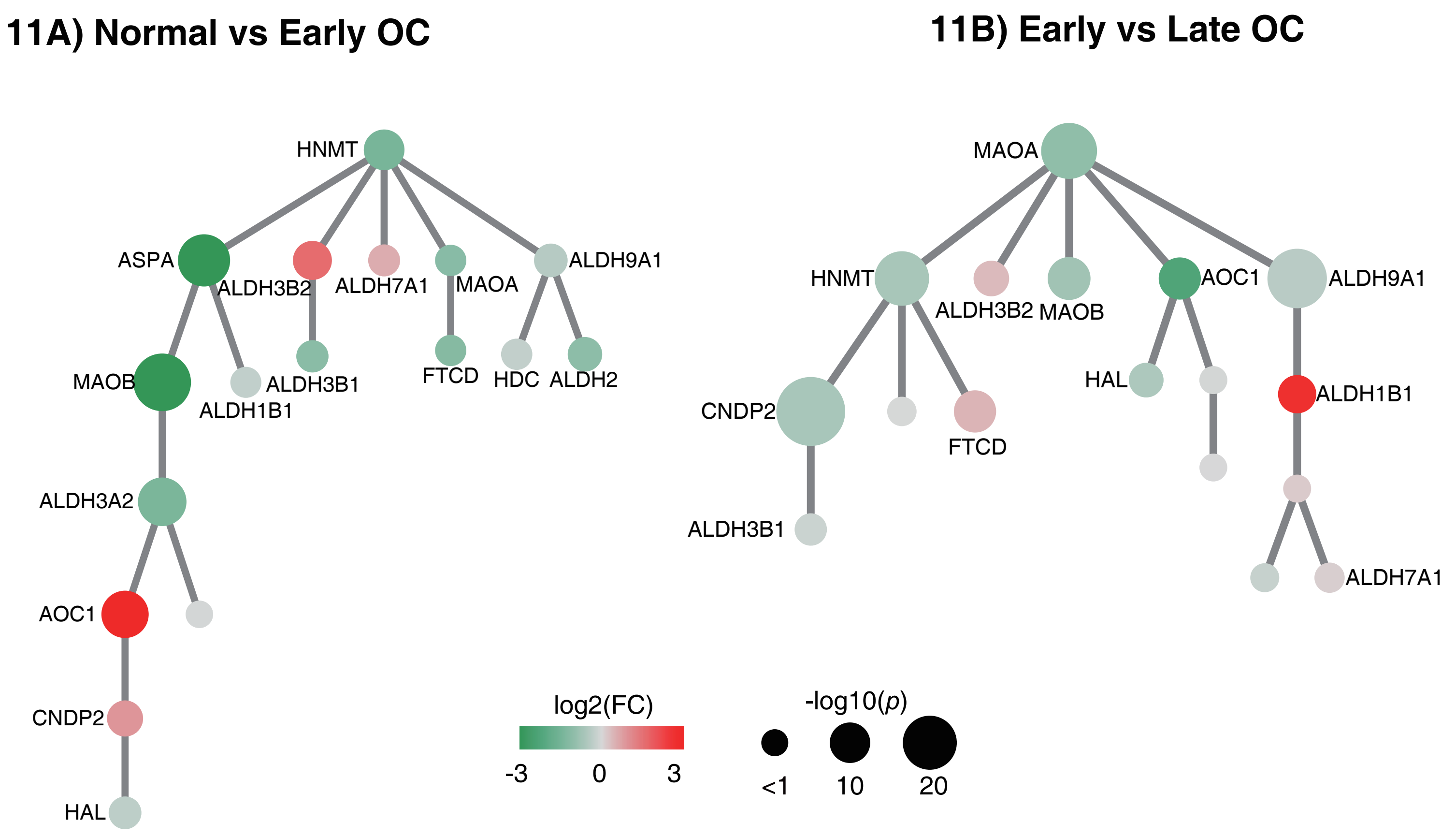

Supplement: Supplementary file 1 — Additional file 1. Gene expression correlation network analysis. [file 12885_2023_11192_MOESM1_ESM.pdf]
