# Supplementary material for: Altered expression of the L-arginine/nitric oxide pathway in ovarian cancer: metabolic biomarkers and biological implications
Source: BMC Cancer. 2023 Sep 8;23:844. doi: 10.1186/s12885-023-11192-8 (PMC10492322; doi:10.1186/s12885-023-11192-8)
Supplement: Supplementary file 2 — Additional file 2: Supplementary 2 Table 1. Gene expression datasets of ovarian cancer tissue studies. Supplementary 2 Figure 1. Quantitative analysis of the targeted L-ARG/NO pathway analytes: arginine and SDMA. (A) Blood analyte abundance in normal, stage I, III, and IV samples. (B) SDMA/ARG ratio as a biomarker to classify different stage cancers from the normal controls. (C) ROC curve of classification. [file 12885_2023_11192_MOESM2_ESM.pptx]

## Slide 1
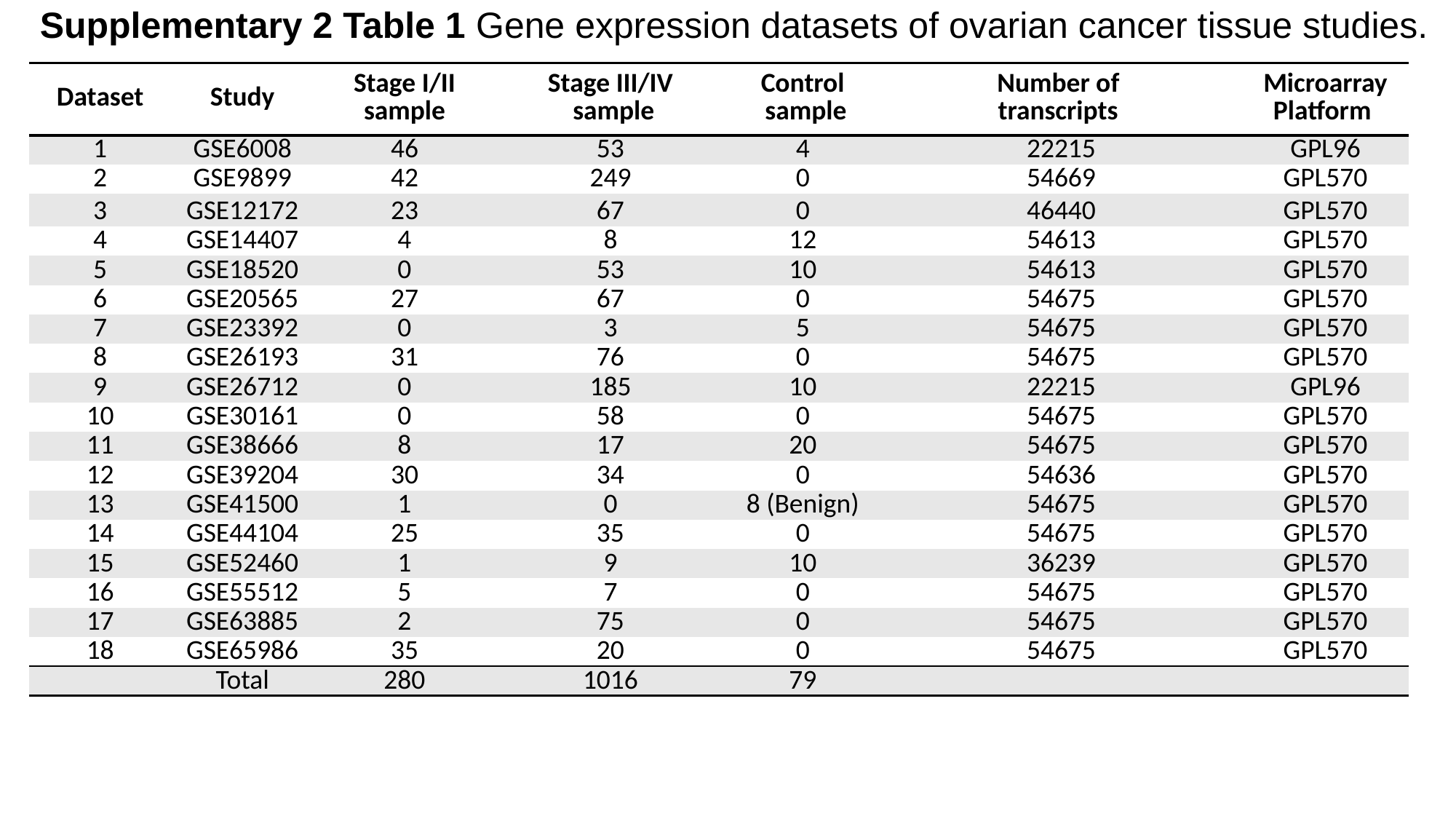

# Supplementary 2 Table 1 Gene expression datasets of ovarian cancer tissue studies.
| Dataset | Study | Stage I/II sample | Stage III/IV sample | Control sample | Number of transcripts | MicroarrayPlatform |
| --- | --- | --- | --- | --- | --- | --- |
| 1 | GSE6008 | 46 | 53 | 4 | 22215 | GPL96 |
| 2 | GSE9899 | 42 | 249 | 0 | 54669 | GPL570 |
| 3 | GSE12172 | 23 | 67 | 0 | 46440 | GPL570 |
| 4 | GSE14407 | 4 | 8 | 12 | 54613 | GPL570 |
| 5 | GSE18520 | 0 | 53 | 10 | 54613 | GPL570 |
| 6 | GSE20565 | 27 | 67 | 0 | 54675 | GPL570 |
| 7 | GSE23392 | 0 | 3 | 5 | 54675 | GPL570 |
| 8 | GSE26193 | 31 | 76 | 0 | 54675 | GPL570 |
| 9 | GSE26712 | 0 | 185 | 10 | 22215 | GPL96 |
| 10 | GSE30161 | 0 | 58 | 0 | 54675 | GPL570 |
| 11 | GSE38666 | 8 | 17 | 20 | 54675 | GPL570 |
| 12 | GSE39204 | 30 | 34 | 0 | 54636 | GPL570 |
| 13 | GSE41500 | 1 | 0 | 8 (Benign) | 54675 | GPL570 |
| 14 | GSE44104 | 25 | 35 | 0 | 54675 | GPL570 |
| 15 | GSE52460 | 1 | 9 | 10 | 36239 | GPL570 |
| 16 | GSE55512 | 5 | 7 | 0 | 54675 | GPL570 |
| 17 | GSE63885 | 2 | 75 | 0 | 54675 | GPL570 |
| 18 | GSE65986 | 35 | 20 | 0 | 54675 | GPL570 |
| | Total | 280 | 1016 | 79 | | |

## Slide 2
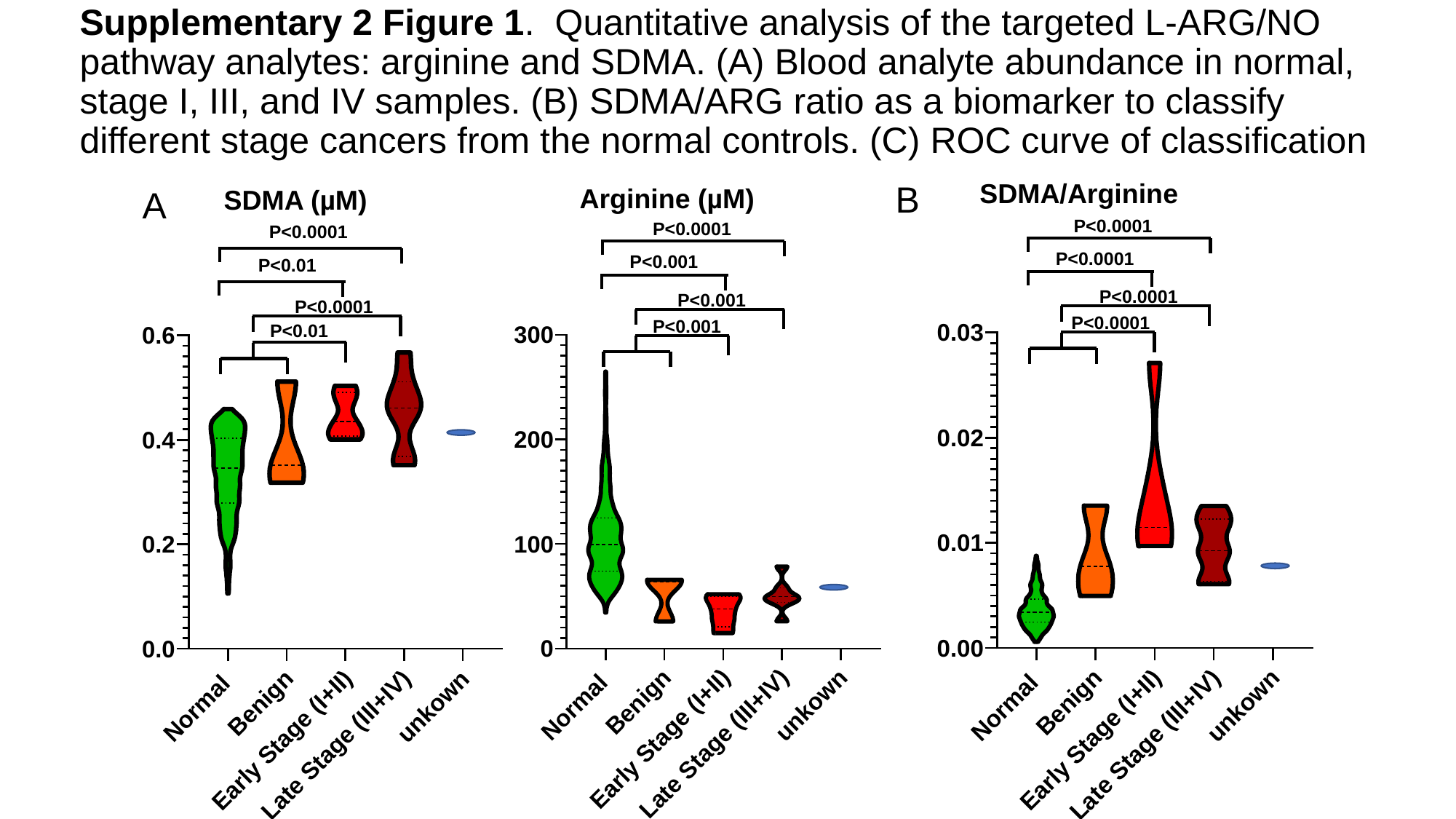

# Supplementary 2 Figure 1. Quantitative analysis of the targeted L-ARG/NO pathway analytes: arginine and SDMA. (A) Blood analyte abundance in normal, stage I, III, and IV samples. (B) SDMA/ARG ratio as a biomarker to classify different stage cancers from the normal controls. (C) ROC curve of classification
SDMA/Arginine
B
Arginine (µM)
A
SDMA (µM)
P<0.0001
P<0.0001
P<0.0001
P<0.0001
P<0.001
P<0.01
P<0.0001
P<0.001
P<0.0001
P<0.0001
P<0.001
P<0.01

## Slide 3
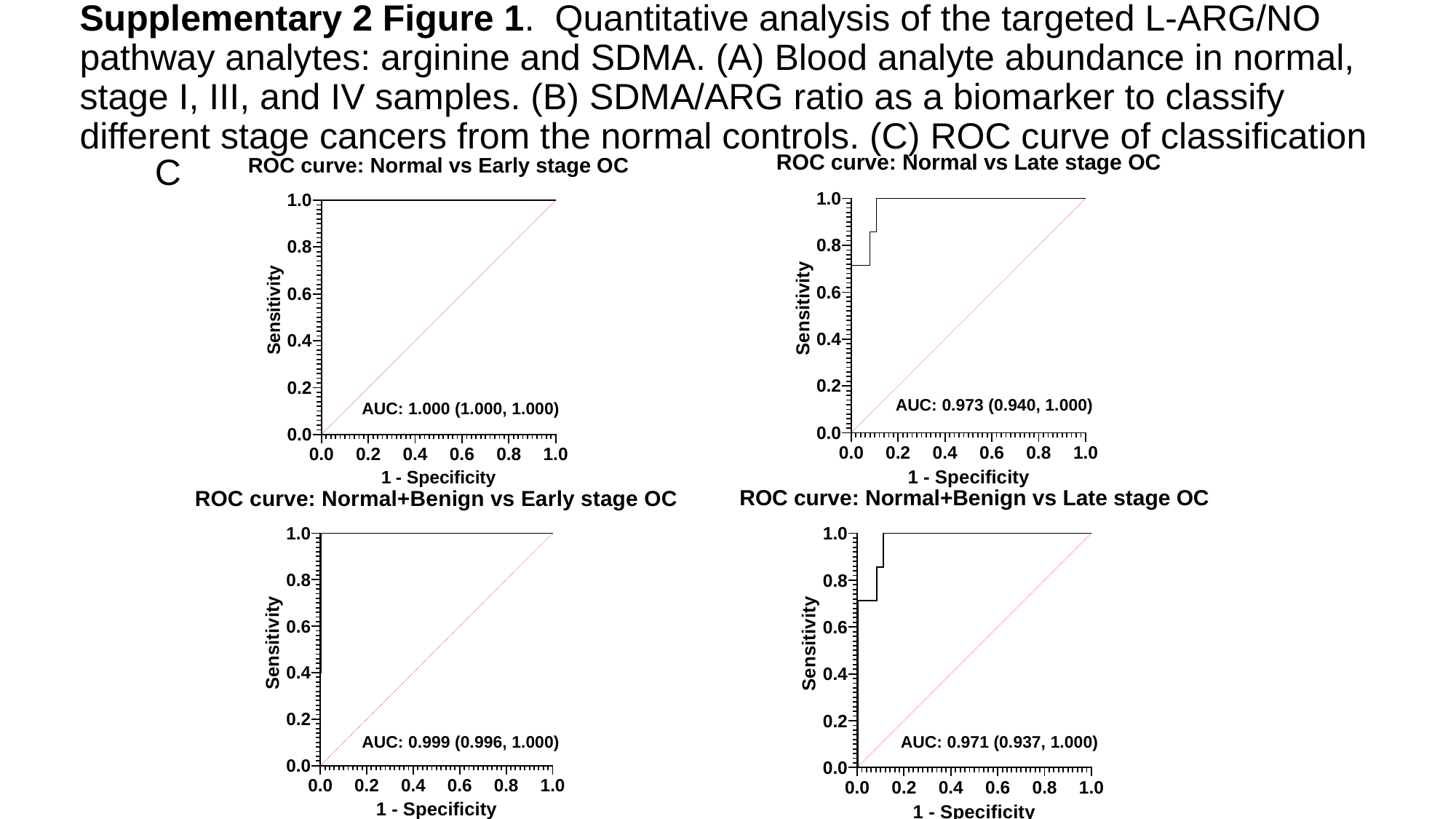

# Supplementary 2 Figure 1. Quantitative analysis of the targeted L-ARG/NO pathway analytes: arginine and SDMA. (A) Blood analyte abundance in normal, stage I, III, and IV samples. (B) SDMA/ARG ratio as a biomarker to classify different stage cancers from the normal controls. (C) ROC curve of classification
C
AUC: 0.973 (0.940, 1.000)
AUC: 1.000 (1.000, 1.000)
AUC: 0.999 (0.996, 1.000)
AUC: 0.971 (0.937, 1.000)
